# Comparative efficacy of different exercise modalities on renal function and glycemic control in patients with non-dialysis diabetic kidney disease: a systematic review and network meta-analysis

**DOI:** 10.3389/fendo.2026.1991940

**Published:** 2026-10-19

**Authors:** Yihang Gong, Jinhui Zheng, Wenrui Huang, Yongkyun Jeon

**Affiliations:** 1 Department of Physical Education, Dankook University, Yongin-si, Gyeonggi-do, Republic of Korea; 2 Winter Olympic College, Harbin Sport University, Harbin, Heilongjiang, China; 3 Shenzhen Hospital of Guangzhou University of Chinese Medicine, Shenzhen, Guangdong, China

**Keywords:** diabetic kidney disease, exercise therapy, glycemic control, network meta-analysis, renal function

## Abstract

**Objective:**

To evaluate the comparative efficacy and safety of structured exercise modalities on renal function and glycemic control in non-dialysis diabetic kidney disease (DKD).

**Design:**

Systematic review and network meta-analysis.

**Data sources:**

Six databases (PubMed, Embase, Cochrane CENTRAL, Web of Science, CNKI, Wanfang) were searched from inception to July 15, 2026.

**Eligibility criteria for selecting studies:**

Randomized controlled trials (RCTs) evaluating structured exercise (aerobic, resistance, combined, or mind–body) versus usual care or an active comparator in adults with non-dialysis DKD (duration ≥4 weeks).

**Outcome measures:**

Renal endpoints were estimated glomerular filtration rate (eGFR) and urinary albumin excretion (UAE); glycemic endpoints were glycated hemoglobin (HbA1c) and fasting blood glucose (FBG).

**Results:**

Twenty RCTs (1,746 patients) were included. Compared with usual care, resistance training (RT) was the sole modality significantly increasing eGFR (mean difference [MD] 11.36 mL/min/1.73 m², 95% CI 4.47 to 18.25; low certainty) and outperforming aerobic training (MD 8.61, 2.22 to 15.00). Mind–body exercise (MBE) was the only intervention significantly reducing UAE (standardized mean difference –1.41, –2.67 to –0.16; low certainty). For glycemic regulation, RT (MD –1.65%), aerobic training (MD –1.07%), and combined training (CT; MD –0.70%) significantly lowered HbA1c (all low certainty). Significant FBG reductions occurred only with RT (MD –1.78 mmol/L; low certainty) and CT (MD –1.71 mmol/L; moderate certainty). In sensitivity analyses, RT’s eGFR benefit lost significance after excluding high-risk trials (MD 8.24, –1.17 to 17.64), and CT’s HbA1c reduction was attenuated in pure-exercise models (MD –0.60%, –1.39 to 0.19). Subgroup analyses showed CT uniquely sustained FBG reductions across short-term (≤12 weeks: MD –1.50) and longer-term (>12 weeks: MD –2.39) regimens. Structured safety was monitored in only five trials (25.0%): three reported mild, transient events without serious harm, while two explicitly declared zero adverse events. Thus, current evidence is insufficient to draw definitive conclusions regarding comparative safety.

**Conclusions:**

Structured exercise confers distinct adaptations in non-dialysis DKD. Combined training provides a reliable foundation for sustained glycemic management, while mind–body exercise represents a viable strategy for albuminuria. Resistance training’s apparent renal benefit warrants caution due to fragility against bias. Prescriptions should be individualized rather than seeking a single optimal modality. Given the sparse monitoring of adverse events, comparative clinical safety cannot be definitively established and mandates routine surveillance.

**Systematic review registration:**

https://www.crd.york.ac.uk/PROSPERO/, identifier CRD420261489091.

## Introduction

Diabetic kidney disease (DKD) represents one of the most common microvascular complications of diabetes and constitutes the primary contributor to chronic kidney disease (CKD) and end-stage kidney disease globally (1). In parallel with the rising prevalence of type 2 diabetes mellitus, the clinical and socioeconomic burden attributable to DKD is expected to increase progressively worldwide (2). Cross-sectional and longitudinal cohorts indicate that 30% to 40% of diabetic individuals eventually exhibit manifestations of renal impairment (1). Concurrently, DKD markedly elevates the incidence of cardiovascular events and premature mortality while accelerating physical functional decline, posing a substantial challenge to healthcare systems.

Exercise therapy is widely recognized as a safe and cost-effective non-pharmacological management strategy for DKD and is endorsed by contemporary clinical practice guidelines, including those from Kidney Disease: Improving Global Outcomes (KDIGO) (3). Its potential benefits are mediated through multiple mechanisms, including improved glycemic stability, reduced oxidative stress and systemic inflammation, and modulation of the renin-angiotensin system (4). Although recent clinical trials have explored the effects of regular exercise on estimated glomerular filtration rate (eGFR) and urinary albumin excretion (UAE), the findings remain inconsistent. Despite a rapidly growing body of evidence, no consensus exists on the optimal exercise modality for improving renal function and glucose metabolism, creating significant uncertainty for clinicians when prescribing personalized exercise regimens.

Earlier meta-analyses have sought to address aspects of this uncertainty but were constrained by important design and methodological caveats. The review by Cai et al. (2021) aggregated physical activity into a single broad category, thereby obscuring potential differences between specific exercise paradigms (5).Conversely, while Nataraj et al. (2023) focused specifically on structured exercise in DKD, their synthesis included only four randomized controlled trials (RCTs) and was limited to standard pairwise comparisons without simultaneous network rankings (6).** A** more recent network meta-analysis by Liu et al. (2024) compared different modalities but pooled a broader non-dialysis CKD population, failing to account for the unique vascular and metabolic features characteristic of DKD (7). Importantly, previous reviews rarely incorporated the Cochrane Risk of Bias 2 (RoB 2) tool or the Confidence in Network Meta-Analysis (CINeMA) framework (8), and none systematically evaluated the potential confounding introduced by co-interventions such as concomitant pharmacotherapy or dietary modifications.

With the recent completion of additional randomized trials, an updated synthesis specifically focused on non-dialysis DKD has become viable. Accordingly, this network meta-analysis was designed to evaluate the comparative effects of four major exercise modalities—aerobic training (AT), resistance training (RT), combined training (CT), and mind-body exercise (MBE)—against usual care on primary renal (eGFR, UAE) and glycemic (HbA1c, FBG) endpoints in adult patients with non-dialysis DKD (CKD stages 1–4 and early diabetic nephropathy). By implementing the Cochrane RoB 2 tool and the CINeMA framework (9), conducting duration-stratified subgroup analyses, and performing sensitivity analyses to isolate pure exercise effects from confounding co-interventions, this network meta-analysis seeks to characterize comparative hierarchies and provide an evidence-graded framework to inform tailored exercise prescriptions.

## Methods

### Study design

This systematic review and network meta-analysis (NMA) was designed and reported in accordance with the Preferred Reporting Items for Systematic Reviews and Meta-Analyses (PRISMA) 2020 statement and the PRISMA extension statement for NMAs (10, 11). The initial literature retrieval was performed up to July 15, 2026, and the protocol was formally registered with PROSPERO (CRD420261489091) on August 27, 2026. *Post-hoc* protocol adjustments and deviations were subsequently implemented to enhance clinical homogeneity and ensure network transitivity: the target population was refined strictly to non-dialysis DKD (formally excluding maintenance dialysis cohorts), the primary analysis was streamlined to four clinically validated closed-loop endpoints (eGFR, UAE, HbA1c, FBG), and exploratory sensitivity scenarios were established. Dropped secondary outcomes (serum creatinine and systolic blood pressure) were relegated to the supplement as exploratory analyses. These *post-hoc* refinements have been documented as transparent protocol amendments in the PROSPERO registry.

### Literature search and study selection

A comprehensive literature search was conducted across six electronic databases: PubMed, Embase, the Cochrane Central Register of Controlled Trials (CENTRAL), Web of Science (Science Citation Index Expanded), China National Knowledge Infrastructure (CNKI), and Wanfang Data, from database inception to July 15, 2026, without language restrictions at the initial search stage. In addition, reference lists of all included randomized controlled trials and relevant prior systematic reviews were manually screened to identify additional potentially eligible studies. Detailed database-specific search strings and boolean queries are documented in **Supplementary Appendix 1** (**Supplementary Tables S1**-**S6**).

All retrieved citations were imported into EndNote reference management software for duplicate removal, which was performed algorithmically and verified manually. Two investigators (YHG and JHZ) independently and in duplicate screened all titles and abstracts against the predefined eligibility criteria. Studies deemed potentially eligible underwent independent full-text assessment. Any disagreements at either screening stage were resolved through consensus discussion or adjudicated by a third senior investigator (YKJ).

### Eligibility criteria

Randomized controlled trials (RCTs) were eligible if they evaluated adult patients (aged ≥18 years) clinically diagnosed with non-dialysis diabetic kidney disease, encompassing CKD stages 1–4 and early diabetic nephropathy, receiving an intervention and follow-up of at least 4 weeks. Eligible trials investigated at least one structured exercise modality—aerobic training (AT), resistance training (RT), combined training (CT), or mind–body exercise (MBE)—against usual clinical care or an active exercise comparator (**Supplementary Table S2.2**). Exercise was required to be the core investigative component; trials involving concurrent supportive co-interventions (e.g., dietary education, nursing models, or pharmacological therapy) were eligible provided that exercise was the defining contrasting factor, with potential confounding from active non-exercise co-interventions systematically evaluated via primary sensitivity analyses. Furthermore, trials were required to report extractable baseline and post-intervention data for at least one primary endpoint: estimated glomerular filtration rate (eGFR), urinary albumin excretion (UAE, assessed via UACR or 24-h UAER), glycated hemoglobin (HbA1c), or fasting blood glucose (FBG).

Trials were excluded if they enrolled patients undergoing maintenance renal replacement therapy or kidney transplantation, or those presenting with non-diabetic kidney diseases, acute kidney injury, or unstable medical conditions contraindicating exercise. In addition, non-randomized studies, observational cohorts, single-arm trials, animal experiments, conference abstracts, and studies with irretrievable data despite author contact were excluded.

### Data extraction and synthesis

Two investigators (YHG and JHZ) independently extracted trial data using a standardized electronic template, with cross-verification and adjudication of discrepancies by a third investigator (YKJ). Extracted data items encompassed: trial and participant characteristics (first author, publication year, country, sample size, age, sex distribution, diabetes duration, and CKD stage); intervention parameters structured by the FITT framework (frequency, intensity, session duration, overall intervention duration, and exercise modalities; **Supplementary Table S2.1**); and documented adverse events. Quantitative outcome extraction was restricted to four primary endpoints: estimated glomerular filtration rate (eGFR), urinary albumin excretion (UAE, assessed via UACR or 24-h UAER), glycated hemoglobin (HbA1c), and fasting blood glucose (FBG).In multi-arm trials involving active pharmacotherapy (e.g., Lin, 2012), we compared the exercise-plus-medication arm with the medication-alone arm to control for background therapy. Interventions incorporating behavioral frameworks to promote adherence (e.g., Cui et al., 2026) were classified by their primary exercise modality (aerobic training).

Primary analyses synthesized post-intervention values (means and standard deviations [SDs] at final follow-up) across treatment groups, adhering to Cochrane methodology for randomized designs where baseline variables are balanced by randomization. For skewed continuous metrics (notably UACR or UAER) or trials presenting data as medians with interquartile ranges (IQRs) or means with 95% confidence intervals, means and SDs were estimated using established mathematical conversion algorithms recommended in the Cochrane Handbook for Systematic Reviews of Interventions (12). In cases of missing or ambiguous data, study authors were contacted; trials with irretrievable data were excluded from quantitative synthesis.

### Quality assessment

Methodological quality of the included randomized trials was independently evaluated by two investigators (YHG and JHZ) using the revised Cochrane risk-of-bias tool for randomized trials (RoB 2) (8). The appraisal evaluated five core methodological domains: bias arising from the randomization process, deviations from intended interventions, missing outcome data, measurement of the outcome, and selection of the reported result. Within each domain, standardized signaling questions informed an algorithm-guided categorization of “low risk of bias”, “some concerns”, or “high risk of bias”. Overall trial-level risk of bias was determined according to the least favorable domain-level assessment, with a trial designated as “high risk” if one or more domains exhibited a high risk of bias. Discrepancies between reviewers were resolved through consensus or formal adjudication by a third investigator (YKJ). Domain-level assessments and overall trial classifications are documented in **Supplementary Table S4**, with trials deemed at high overall risk of bias subsequently evaluated in sensitivity analyses.

### Statistical analysis

Quantitative evidence synthesis was performed within a frequentist network meta-analysis framework using the network and mvmeta packages in Stata (version 17.0; StataCorp LLC). To account for expected clinical diversity in exercise prescriptions and patient baseline characteristics, all network models were fitted using a restricted maximum likelihood (REML) random-effects model. Continuous treatment effects were expressed as mean differences (MDs) with 95% confidence intervals (CIs) for eGFR, HbA1c, and FBG, whereas standardized mean differences (SMDs) with 95% CIs were calculated for UAE to harmonize disparate reporting units (UACR in mg/g vs. 24-h UAER in mg/24h). Both head-to-head exercise trials and usual-care-controlled trials were integrated simultaneously into the network geometry via standard inverse-variance weighting without differential treatment.

The transitivity assumption was evaluated both qualitatively (by comparing eligibility criteria and study protocols) and quantitatively (by calculating pooled, sample-size-weighted means for clinical effect modifiers—age, sex ratio, baseline glycemic status, and follow-up duration—across treatment comparisons; **Supplementary Table S8.1**). Statistical heterogeneity was quantified across the network using the global heterogeneity variance parameter τ², alongside Cochran’s Q test and the I² statistic for pairwise contrasts, with P < 0.10 or I² > 50% denoting substantial heterogeneity (13). Global network consistency was examined using the design-by-treatment interaction model. Local inconsistency was evaluated using the node-splitting method, which was strictly restricted to comparisons forming closed loops in the network geometry. In the absence of statistically significant incoherence (P > 0.05), the consistency model was retained for primary inference.

Treatment hierarchies were characterized using the surface under the cumulative ranking curve (SUCRA) and mean ranks (14), where higher values denote a greater cumulative probability of superior efficacy. To avoid clinical misinterpretation driven solely by ranking probabilities, SUCRA hierarchies were interpreted as exploratory and evaluated alongside relative effect estimates and CINeMA certainty ratings. Small-study effects and potential publication bias were visually examined using comparison-adjusted funnel plots (15), which were constructed across all four endpoints as each was informed by at least 10 trials.

### Subgroup and sensitivity analyses

Subgroup analyses were conducted to evaluate potential time-dependent treatment effect modification by stratifying trials according to intervention duration: short-term (≤12 weeks) versus longer-term (>12 weeks) regimens (**Supplementary Table S11.1**). This stratification sought to determine whether renal and glycemic adaptations differed between acute physiological responses and sustained training exposures.

To rigorously assess model robustness and dissect potential sources of methodological and clinical heterogeneity, three sensitivity analyses were implemented (**Supplementary Figure S10.1**). First, trials evaluated as having an overall high risk of bias under the Cochrane RoB 2 tool were excluded (n=2 excluded, leaving 18 RCTs; **Supplementary Table S10.1**) to appraise the impact of methodological quality. Second, under a strict pure-exercise scenario, all trials with active non-exercise co-interventions—including oral pharmacological therapy (traditional Chinese medicine), specialized nursing protocols, and structured nutritional guidance—were removed (n=4 excluded, leaving 16 RCTs; **Supplementary Table S10.2**) to isolate the unconfounded therapeutic effects of exercise. Third, under a pragmatic clinical scenario, trials involving active pharmacological agents or specialized nursing models were removed while intentionally retaining guideline-directed dietary and nutritional guidance (n=2 excluded, leaving 18 RCTs; **Supplementary Table S10.3**). Because dietary management represents an intrinsic component of standard medical care in DKD, this contrast evaluated whether routine nutritional counseling confounded exercise stability or whether treatment hierarchies persisted in real-world lifestyle management paradigms. Across all scenarios, network models were re-estimated, with findings considered robust if the direction, magnitude, and statistical inferences of effect sizes remained consistent with primary analyses.

### Certainty of evidence assessment

The certainty of evidence for all network comparisons was systematically evaluated using the Confidence in Network Meta-Analysis (CINeMA) framework. Two investigators (YHG and JHZ) independently conducted the assessments via the dedicated web application (https://cinema.ispm.unibe.ch), resolving any discrepancies through adjudication with a third investigator (YKJ). CINeMA grades evidence across six core methodological domains: within-study bias (informed by relative contributions of study-level RoB 2 evaluations), reporting bias (appraised via comprehensive search scrutiny and comparison-adjusted funnel plot symmetry), indirectness (relevance of trial populations, interventions, and outcome definitions to the target research question), imprecision (assessed primarily by whether the 95% confidence intervals crossed the line of no effect, given the lack of universally established minimal clinically important differences across all endpoints in this specific cohort), heterogeneity (variability across direct pairwise contrasts and prediction intervals), and incoherence (agreement between direct and indirect evidence in closed loops alongside global test statistics). Each domain was categorized as having “no concerns”, “some concerns”, or “major concerns”, which were subsequently integrated to grade the overall certainty of evidence as “high”, “moderate”, “low”, or “very low”. Detailed domain-level judgments and CINeMA profile evidence summaries across all network contrasts are documented in **Supplementary Appendix 8**.

## Results

### Study selection

Of 783 records identified from database retrieval and citation tracking, 607 remained after duplicate removal. Screening of titles and abstracts identified 51 reports for full-text eligibility appraisal. Following full-text evaluation, 31 studies were excluded—predominantly owing to maintenance dialysis, non-qualifying co-interventions, or unextractable outcome data (**Figure 1**). Ultimately, 20 randomized controlled trials evaluating non-dialysis diabetic kidney disease met all inclusion criteria and were incorporated into the network meta-analysis (16–35).

**Figure 1:**
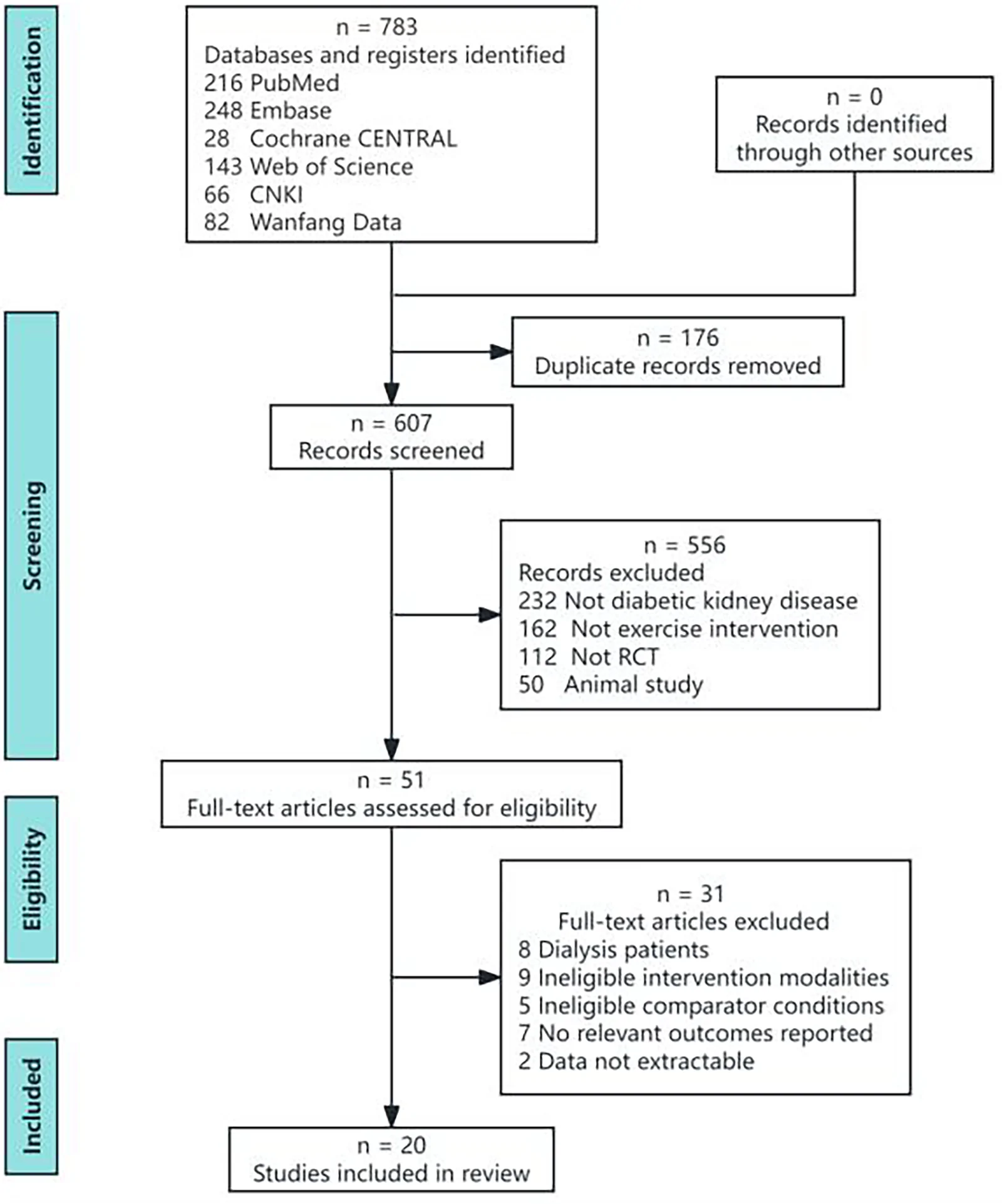
Flow diagram of the study selection process.

### Study characteristics

Twenty RCTs published between 2009 and 2026, comprising 1,746 adult participants with non-dialysis DKD, met all eligibility criteria (**Table 1**; **Supplementary Table S2.1**). Trials were predominantly conducted in China (n=14), alongside cohorts from the United States, India, Japan, France/Belgium, and Iran. Sample sizes ranged from 13 to 260 participants. Baseline renal status comprised early diabetic nephropathy (EDN, Mogensen stage III) in 12 trials and CKD stages 1–4 in eight trials, with no trials enrolling dialysis-dependent cohorts. Intervention durations spanned 4 to 104 weeks (median: 12 weeks). The five-node network incorporated four structured exercise modalities (AT, RT, CT, MBE) and usual care (UC), comprising 17 usual-care-controlled trials and three active head-to-head comparisons (RT vs. AT, n=2; CT vs. AT, n=1). Across the network, extractable data were available for HbA1c (n = 17 trials), FBG (n = 14), eGFR (n = 13), and UAE (n = 12). Detailed exercise prescriptions structured under the FITT framework are documented in **Supplementary Table S2.1**.Structured safety was documented in five trials (25.0%), reporting mild events or explicitly confirming zero adverse events, with no exercise-related serious adverse events documented.

**Table 1:** Baseline characteristics.

Study (Year)	Sample size (I/C)	Age, years (I/C)	Clinical stage	Intervention	Duration (weeks)	Comparator
Leehey 2016	18/18	65.4 ± 8.7/66.6 ± 7.5	CKD 2-4	CT	52	UC
Leehey 2009	7/6	66 (median)	CKD 2-4	AT	24	UC
Nataraj 2024	30/30	59.0 ± 7.6/58.3 ± 7.8	CKD 1-4	CT	24	UC
Waki 2024	66/60	58.6 ± 10.1/60.3 ± 8.7	CKD 1-3	AT	48	UC
Hadjadj 2026	59/44	66.2 ± 8.5/65.9 ± 6.7	CKD 1-3	CT	104	UC
Dong 2019	60/61	56 ± 14.1 (overall)	CKD 2-3	AT	24	UC
Moghadasi 2022	15/15	52.8 ± 7.3 (overall)	CKD 2-4	RT	8	UC
Li 2024*	30/30	36.6 ± 13.2/37.0 ± 13.0	EDN	RT	12	AT
Lin 2012	30/30	60 ± 2.2/60 ± 2.2	EDN	MBE	12	UC
Liu 2020	38/37	62.2 ± 3.8/63.1 ± 4.2	EDN	CT	12	UC
Cui 2026	46/47	NR	EDN	AT	12	UC
Sun 2018	33/33	50.6 ± 8.2/51.8 ± 9.8	EDN	MBE	12	UC
Li 2025	90/90	58.3 ± 11.9/57.4 ± 11.4	EDN	CT	8	UC
Kong 2017*	30/30	48.3 ± 10.7/49.1 ± 9.3	EDN	RT	24	AT
Tong 2026	52/52	69.9 ± 4.3/70.1 ± 4.6	CKD 2-3	CT	12	UC
Zhong 2023*	52/51	54.1 ± 7.8/53.7 ± 7.9	EDN	CT	8	AT
Tian 2026	130/130	63.0 ± 8.2/62.9 ± 7.0	EDN	MBE	24	UC
Wu 2024	30/30	55.0 ± 8.9/58.1 ± 9.4	EDN	CT	24	UC
Wu 2025	25/25	67.0 ± 7.5/66.0 ± 8.5	EDN	AT	4	UC
Fang 2025	43/43	60.9 ± 3.6/59.8 ± 3.8	EDN	RT	12	UC

AT, aerobic training; CKD, chronic kidney disease; CT, combined training (aerobic plus resistance); EDN, early diabetic nephropathy; I/C, intervention group/comparator group; MBE, mind–body exercise; NR, not reported; RT, resistance training; UC, usual care.

* Head-to-head comparison trial between active exercise modalities.

### Risk of bias in included studies

Methodological quality across the 20 included RCTs was appraised using the Cochrane RoB 2 tool (**Figure 2**; **Supplementary Table S4**). Overall, 10 trials (50.0%) were judged to be at low risk of bias, eight (40.0%) raised some concerns, and two (10.0%) exhibited high risk of bias due to high attrition rates (Leehey 2016 and Moghadasi 2022). Outcome measurement and randomization processes were judged to be at low risk across all included trials. Comparison-adjusted funnel plots revealed no evident asymmetry or substantial small-study effects across the four evaluated endpoints (**Supplementary Appendix 9**; **Supplementary Figures S9.1**-**S9.4**).

**Figure 2:**
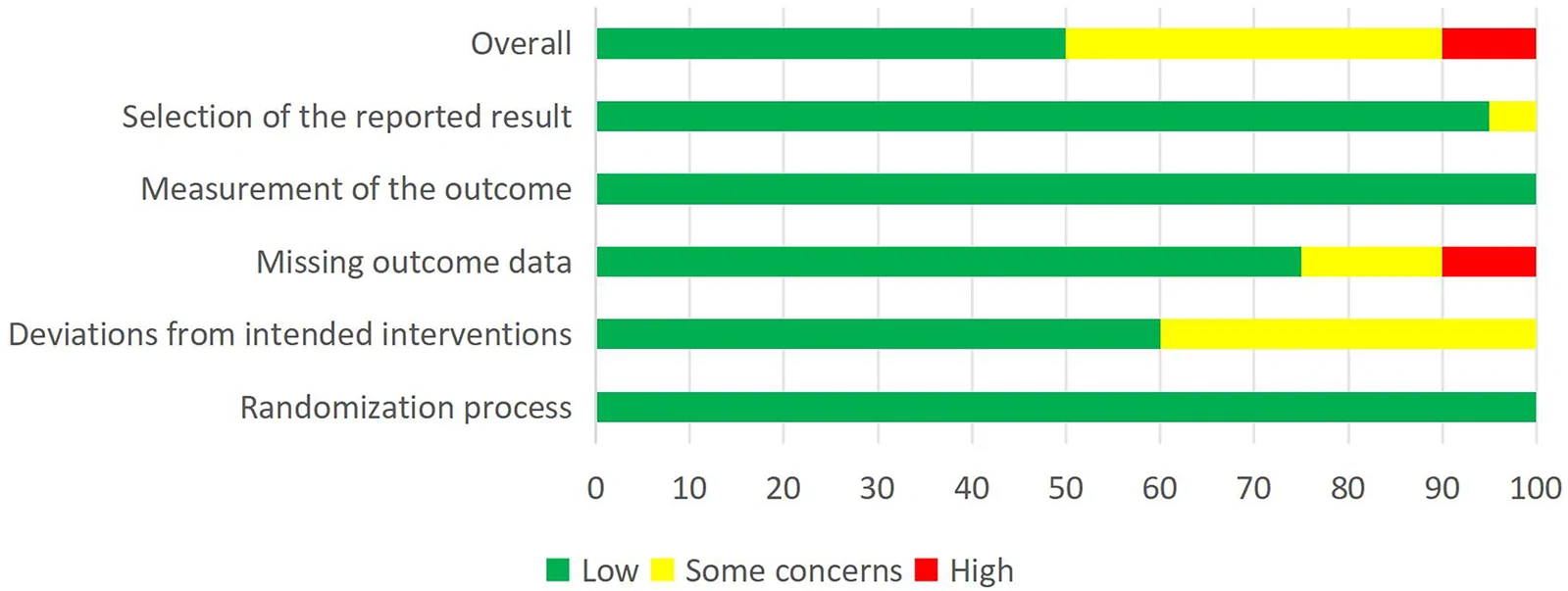
Risk of bias summary(presented as percentages).

### Network geometry, heterogeneity, and inconsistency

Global heterogeneity across the networks was quantified using the between-study variance parameter (τ² = 13.12 for eGFR, 0.73 for UAE, 0.55 for HbA1c, and 0.69 for FBG; **Supplementary Table S5.1**). Global consistency testing via the design-by-treatment interaction model indicated no overall inconsistency across any evaluated endpoint (all P > 0.05). In closed geometric loops, node-splitting analyses confirmed agreement between direct and indirect comparisons, with no statistically significant incoherence identified (loop-specific P-values: 0.054 to 0.864; **Supplementary Tables S5.2**-**S5.5**).

### Primary outcomes

#### eGFR

The eGFR network incorporated 13 trials involving 935 participants across four interventions (AT, CT, RT, and UC), with UC serving as the reference comparator (**Figure 3**; **Supplementary Figure S8.1**). Compared with UC, only RT produced a statistically significant increase in eGFR (MD 11.36 mL/min/1.73 m², 95% CI 4.47 to 18.25), whereas CT and AT showed no statistically significant differences (**Figure 3**). In head-to-head comparisons, RT demonstrated a statistically significant improvement over AT (MD 8.61 mL/min/1.73 m², 95% CI 2.22 to 15.00), with no significant differences observed between the remaining modalities (**Supplementary Table S7.1**). SUCRA values were 98.8% for RT, 55.2% for CT, 41.2% for AT, and 4.8% for UC (**Supplementary Figure S6.1**; **Supplementary Table S6.1**). According to the CINeMA framework, certainty of evidence was rated as low across all comparisons(**Supplementary Table S8.2**).

**Figure 3:**
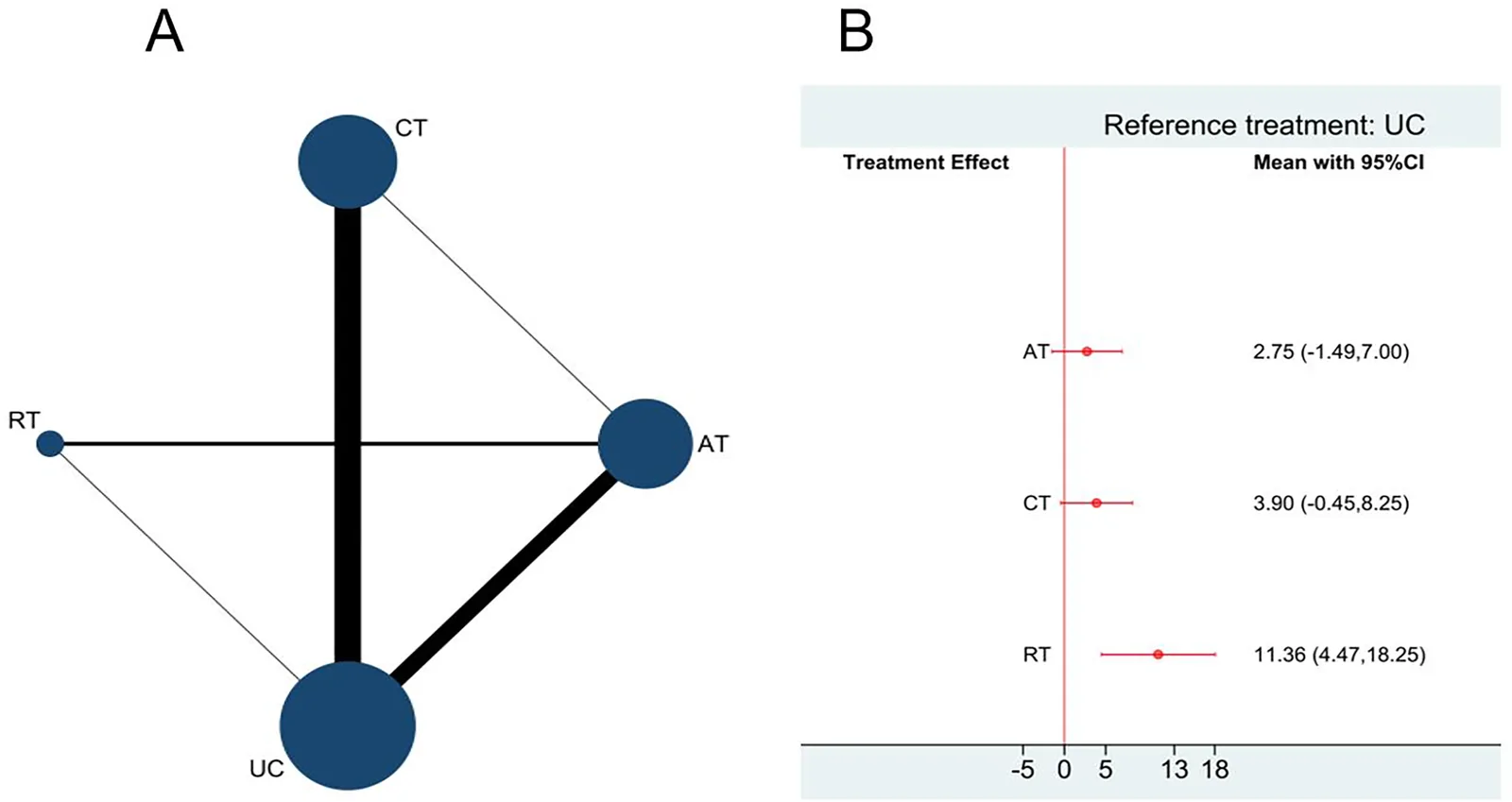
Network plot **(A)** and forest plot **(B)** for eGFR. In panel **(B)**, a mean difference (MD) > 0 indicates a beneficial effect of the exercise modality compared with usual care (UC). Node sizes reflect sample sizes, and line thicknesses represent the number of comparison trials. AT, aerobic training; CI, confidence interval; CT, combined training(aerobic plus resistance); eGFR, estimated glomerular filtration rate; MD, mean difference; RT, resistance training; UC, usual care.

#### UAE

The UAE network incorporated 12 trials involving 966 participants across five interventions (AT, CT, MBE, RT, and UC), with UC serving as the reference comparator (**Figure 4**; **Supplementary Figure S8.3**). Compared with UC, only MBE produced a statistically significant reduction in UAE (SMD –1.41, 95% CI –2.67 to –0.16), whereas RT, AT, and CT showed no statistically significant differences (**Figure 4**). In head-to-head comparisons among active exercise modalities, no statistically significant differences were observed across any comparisons (**Supplementary Table S7.2**). SUCRA values were 80.3% for RT, 77.8% for MBE, 47.6% for AT, 40.3% for CT, and 4.0% for UC (**Supplementary Figure S6.2**; **Supplementary Table S6.2**). According to the CINeMA framework, certainty of evidence was rated as low across all comparisons (**Supplementary Table S8.3**).

**Figure 4:**
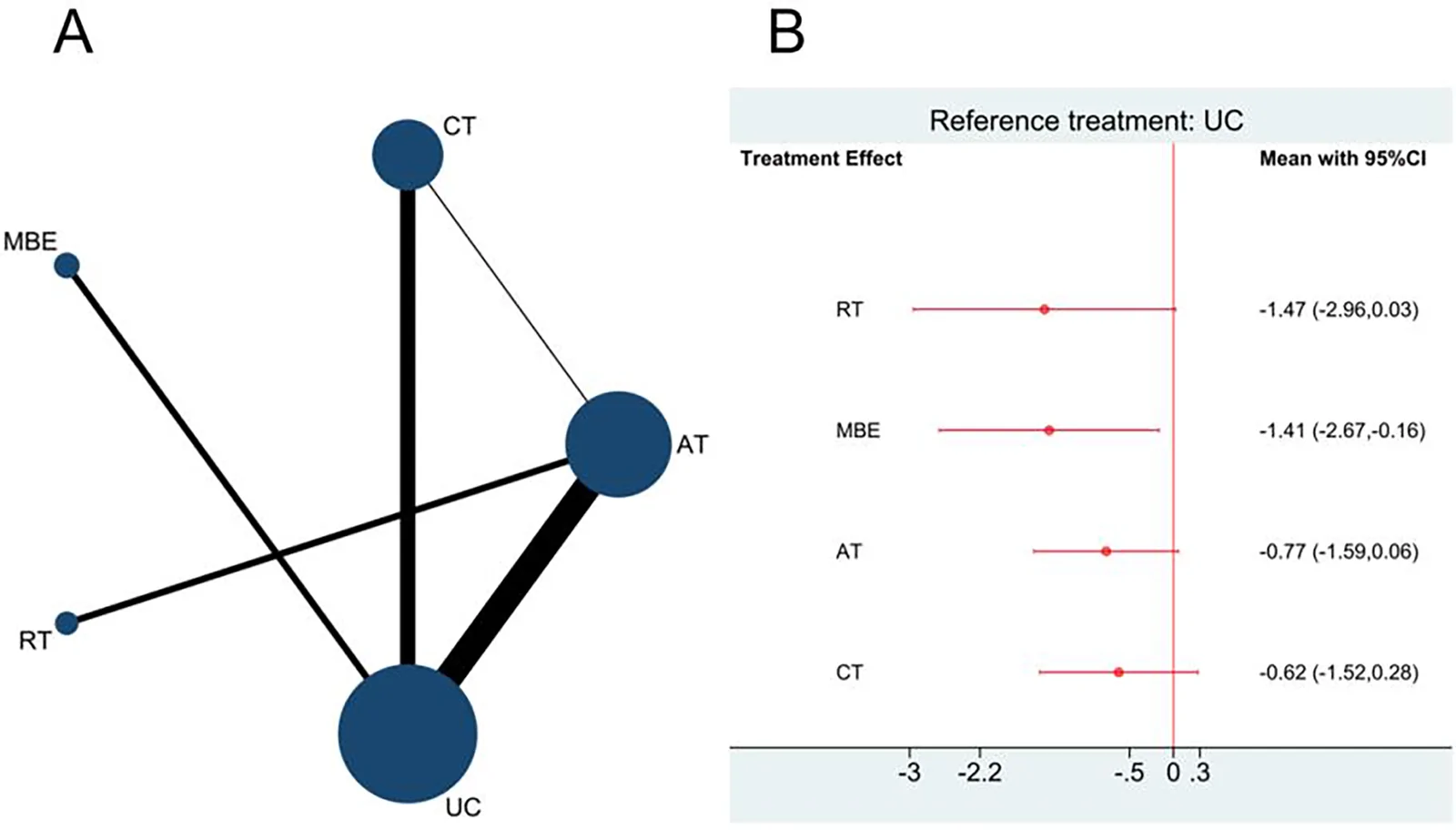
Network plot **(A)** and forest plot **(B)** for UAE. In panel **(B)**, a standardized mean difference (SMD) < 0 indicates a beneficial effect of the exercise modality in reducing UAE compared with usual care (UC). AT, aerobic training; CI, confidence interval; CT, combined training; MBE, mind–body exercise; RT, resistance training; SMD, standardized mean difference; UAE, urinary albumin excretion; UC, usual care.

#### HbA1c

The HbA1c network incorporated 17 trials involving 1,570 participants across five interventions (AT, CT, MBE, RT, and UC), with UC serving as the reference comparator (**Figure 5**; **Supplementary Figure S8.5**). Compared with UC, statistically significant reductions in HbA1c were produced by RT (MD –1.65%, 95% CI –2.67 to –0.62), AT (MD –1.07%, 95% CI –1.71 to –0.43), and CT (MD –0.70%, 95% CI –1.33 to –0.08), whereas MBE showed no statistically significant difference (**Figure 5**; **Supplementary Table S7.3**). In head-to-head comparisons among active exercise modalities, no statistically significant differences were observed across any comparisons (**Supplementary Table S7.3**). SUCRA values were 94.2% for RT, 70.1% for AT, 47.9% for CT, 31.3% for MBE, and 6.5% for UC (**Supplementary Figure S6.3**; **Supplementary Table S6.3**). According to the CINeMA framework, certainty of evidence was rated as low across all comparisons (**Supplementary Table S8.4**).

**Figure 5:**
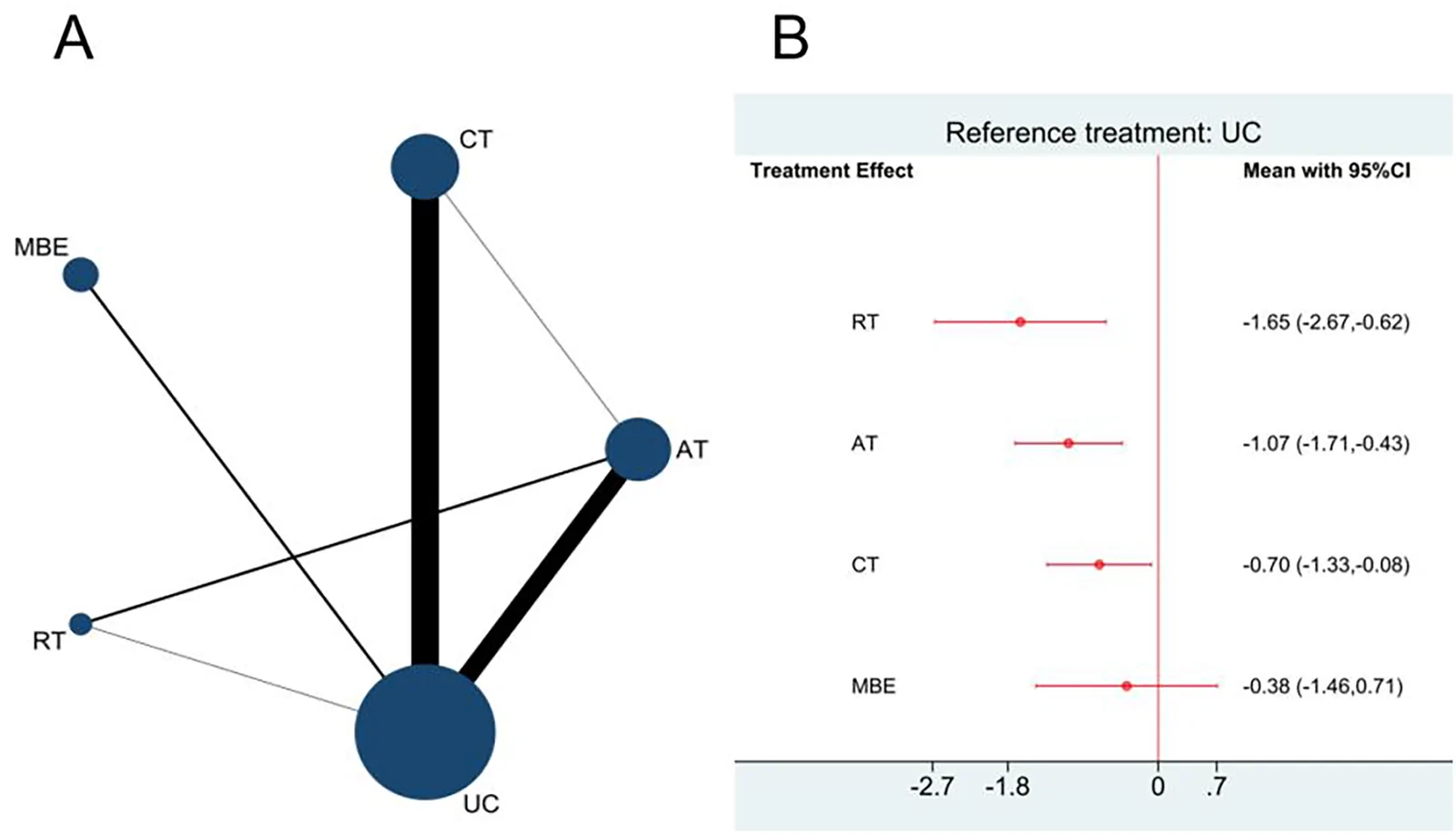
Network plot **(A)** and forest plot **(B)** for HbA1c. In panel **(B)**, a mean difference (MD) < 0 indicates a beneficial effect of the exercise modality in reducing HbA1c (%) compared with usual care (UC). AT, aerobic training; CI, confidence interval; CT, combined training; HbA1c, glycated hemoglobin; MBE, mind–body exercise; MD, mean difference; RT, resistance training; UC, usual care.

#### FBG

The FBG network incorporated 14 trials involving 1,410 participants across five interventions (AT, CT, MBE, RT, and UC), with UC serving as the reference comparator (**Figure 6**; **Supplementary Figure S8.7**). Compared with UC, statistically significant reductions in FBG were produced by RT (MD –1.78 mmol/L, 95% CI –2.92 to –0.64) and CT (MD –1.71 mmol/L, 95% CI –2.51 to –0.91), whereas MBE and AT showed no statistically significant differences (**Figure 6**). In head-to-head comparisons among active exercise modalities, both RT (MD –1.87 mmol/L, 95% CI –3.16 to –0.58) and CT (MD –1.80 mmol/L, 95% CI –2.85 to –0.76) demonstrated statistically significant reductions in FBG compared with AT, with no significant differences observed between the remaining exercise modalities (**Supplementary Table S7.4**). SUCRA values were 86.1% for RT, 84.0% for CT, 47.2% for MBE, 17.9% for AT, and 16.8% for UC (**Supplementary Figure S6.4**; **Supplementary Table S6.4**). According to the CINeMA framework, certainty of evidence was rated as moderate for CT versus UC and CT versus AT, and low for all remaining comparisons (**Supplementary Table S8.5**).

**Figure 6:**
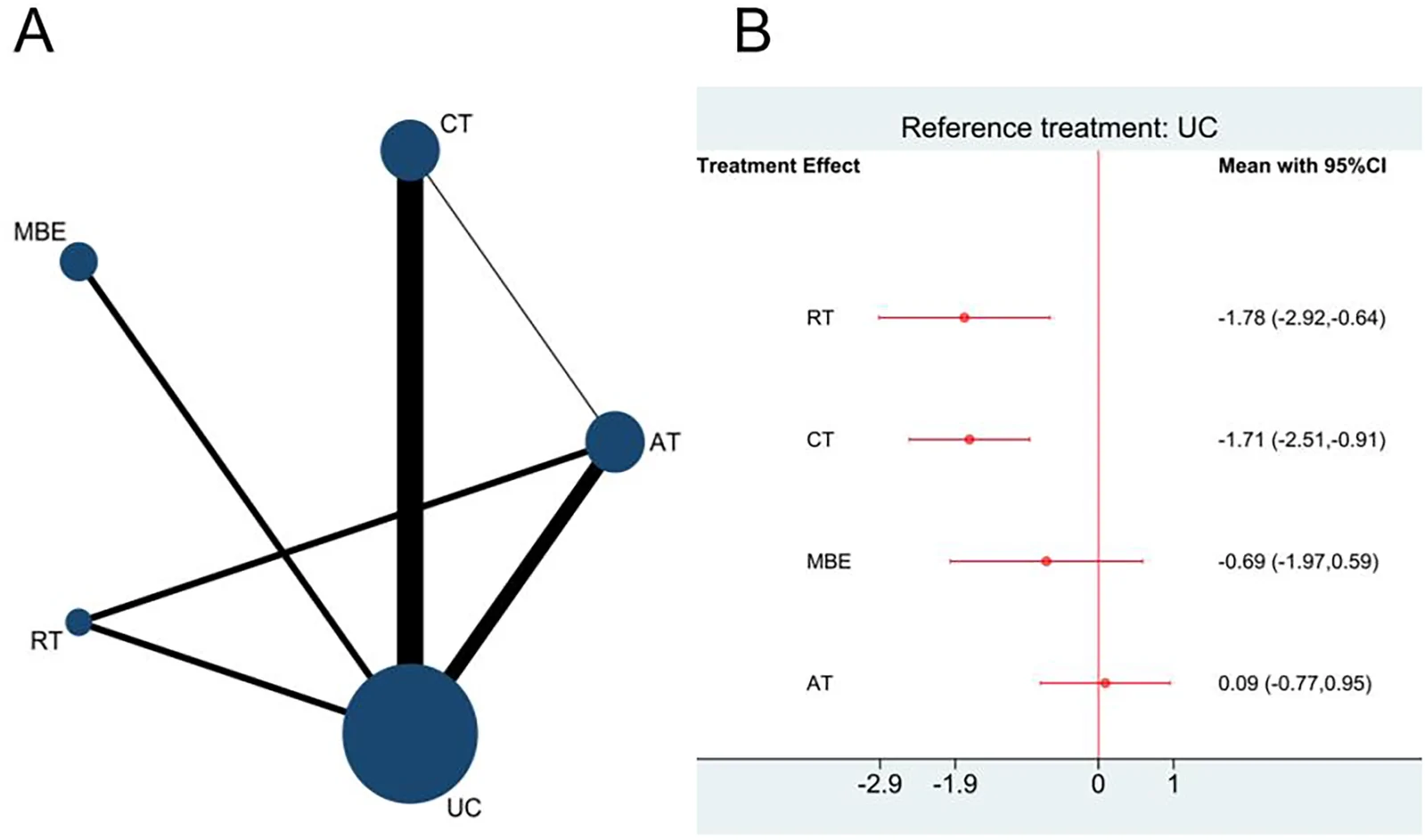
Network plot **(A)** and forest plot **(B)** for FBG. In panel **(B)**, a mean difference (MD) < 0 indicates a beneficial effect of the exercise modality in reducing FBG (mmol/L) compared with usual care (UC). AT, aerobic training; CI, confidence interval; CT, combined training; FBG, fasting blood glucose; MBE, mind–body exercise; MD, mean difference; RT, resistance training; UC, usual care.

### Summary of treatment rankings

To provide a visual summary of the comparative hierarchies among distinct exercise modalities, a SUCRA sunburst heat map was constructed for the four primary endpoints (**Figure 7**). Based on cumulative ranking probabilities, RT yielded the highest nominal SUCRA values for improving eGFR (98.8%), UAE (80.3%), HbA1c (94.2%), and FBG (86.1%), with MBE demonstrating the second highest probability for UAE (77.8%). CT ranked second for both FBG (84.0%) and eGFR (55.2%), while UC consistently ranked lowest across all evaluated outcomes.

**Figure 7:**
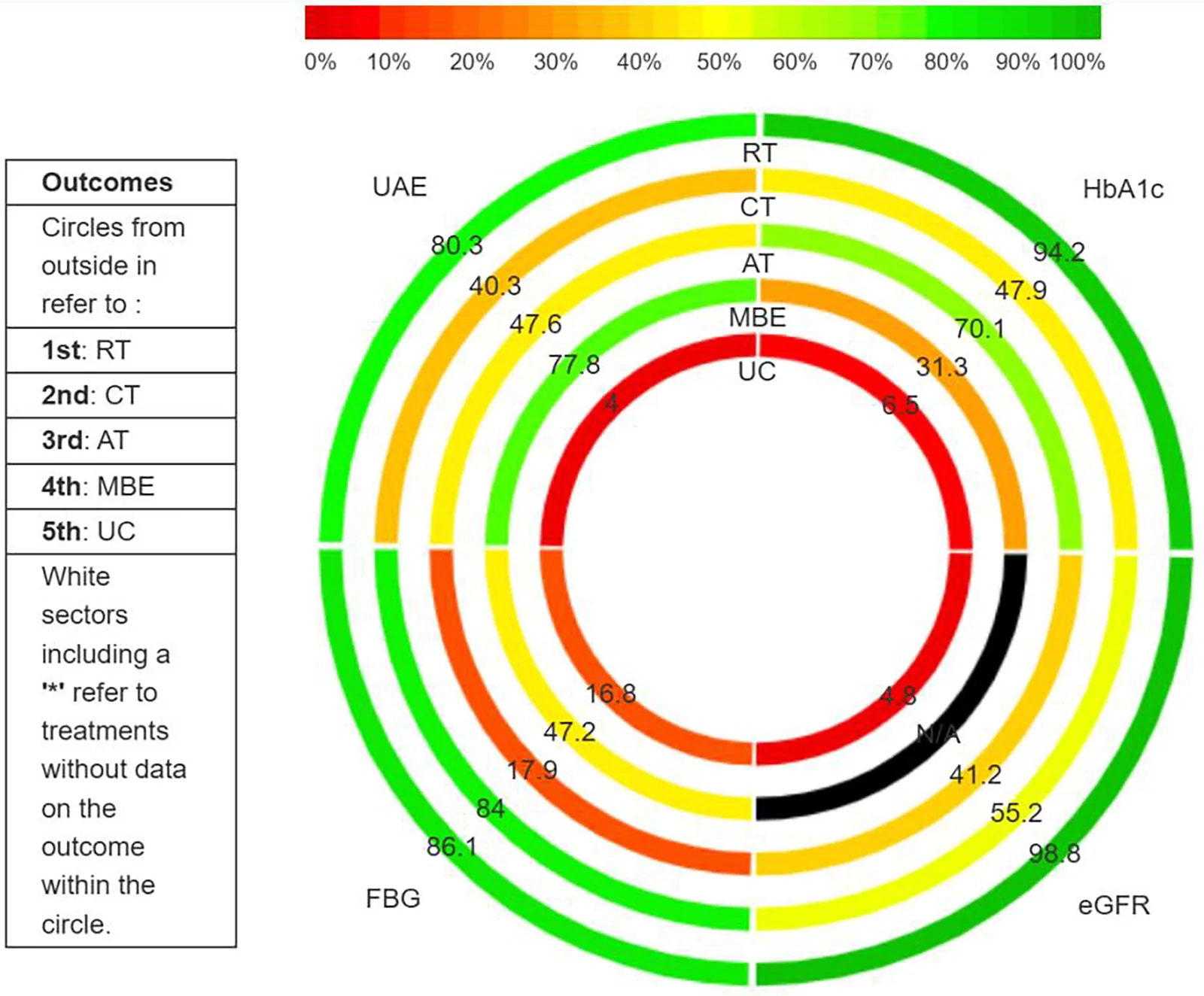
SUCRA heat map for primary outcomes. Colors represent the Surface Under the Cumulative Ranking curve (SUCRA) probabilities (0% to 100%), with greener shades indicating a higher probability of being the most effective intervention. Specific ring designations and missing data indicators are detailed in the embedded legend. Abbreviations: AT, aerobic training; CT, combined training; eGFR, estimated glomerular filtration rate; FBG, fasting blood glucose; HbA1c, glycated hemoglobin; MBE, mind–body exercise; RT, resistance training; SUCRA, surface under the cumulative ranking curve; UAE, urinary albumin excretion; UC, usual care.

### Sensitivity analyses

Three primary sensitivity analyses were performed according to the evaluation framework (**Supplementary Figure S10.1**). Following the exclusion of two trials rated at high risk of bias, effect directions across all outcomes remained consistent with the primary analysis, although the increase in eGFR with RT lost statistical significance (MD 8.24 mL/min/1.73 m², 95% CI –1.17 to 17.64) and the reduction in UAE with RT achieved statistical significance (MD –1.52, 95% CI –3.02 to –0.02) (**Supplementary Table S10.**1). When restricting the synthesis to pure exercise interventions by excluding four trials with potential confounding co-interventions, the reduction in HbA1c with CT lost statistical significance (MD –0.60%, 95% CI –1.39 to 0.19), whereas statistical inferences for all remaining comparisons across eGFR, UAE, and FBG remained concordant with the primary analysis (**Supplementary Table S10.2**). In the pragmatic scenario excluding trials involving active medication and specialized nursing models while retaining nutritional guidance, statistical conclusions for all exercise modalities across the four endpoints were identical to the primary analysis (**Supplementary Table S10.3**).

### Subgroup analyses

Subgroup analyses were conducted according to intervention duration (≤12 weeks vs >12 weeks; **Supplementary Table S11.1**). For eGFR, statistically significant increases were observed in the short-term subgroup (≤12 weeks) following RT (MD 11.94 mL/min/1.73 m², 95% CI 5.95 to 17.93) and AT (MD 3.82 mL/min/1.73 m², 95% CI 0.09 to 7.55), whereas no exercise modalities reached statistical significance in the long-term subgroup (>12 weeks) (**Supplementary Table S11.1**). For UAE, statistically significant reductions were produced in the short-term subgroup by RT (SMD –1.41, 95% CI –2.24 to –0.57) and CT (SMD –0.96, 95% CI –1.85 to –0.07), while no modalities showed statistically significant effects in the long-term subgroup (**Supplementary Table S11.1**). For HbA1c, statistically significant reductions were achieved in the short-term subgroup by RT (MD –1.58%, 95% CI –2.72 to –0.43), AT (MD –1.21%, 95% CI –2.03 to –0.38), and CT (MD –1.06%, 95% CI –1.80 to –0.31), whereas no modalities retained statistical significance in the long-term subgroup (**Supplementary Table S11.1**). For FBG, statistically significant reductions were maintained across both short- and long-term subgroups by RT (≤12 weeks: MD –1.73 mmol/L, 95% CI –3.38 to –0.09; >12 weeks: MD –2.76 mmol/L, 95% CI –4.76 to –0.76) and CT (≤12 weeks: MD –1.50 mmol/L, 95% CI –2.77 to –0.23; >12 weeks: MD –2.39 mmol/L, 95% CI –3.74 to –1.04) (**Supplementary Table S11.1**).

### Adverse events and safety

Structured safety and adverse event monitoring was documented in five of the 20 included trials (25.0%). Among these, two trials explicitly confirmed the absence of any intervention-related adverse events (Leehey et al., 2016; Waki et al., 2024), while three trials reported specific mild, transient events—including transient hypoglycemia, musculoskeletal discomfort, minor gastrointestinal symptoms, and falls without serious physical harm (Hadjadj et al., 2026; Li et al., 2024; Tian et al., 2026). The remaining 15 trials (75.0%) completely omitted safety surveillance. Consequently, empirical data are insufficient to quantitatively synthesize comparative safety profiles across modalities.

## Discussion

### Principal findings

This network meta-analysis of 20 randomized controlled trials involving 1,746 patients with non-dialysis diabetic kidney disease provides a comprehensive comparative evaluation of four structured exercise modalities (aerobic, resistance, combined, and mind–body exercise) across primary renal and glycemic outcomes. Overall, structured exercise yielded modality-dependent benefits across renal parameters and glycemic control. Safety reporting was severely sparse, documented in only five trials (25.0%) with no exercise-attributable serious adverse events; however, this substantial underreporting precludes definitive conclusions regarding universal clinical tolerability.

Distinct comparative patterns emerged across physiological targets. For renal endpoints, resistance training was the sole modality to produce a statistically significant increase in eGFR compared with usual care, while also demonstrating significant superiority over aerobic training. Conversely, mind–body exercise was the only intervention yielding a statistically significant reduction in urinary albumin excretion. For glycemic regulation, resistance, aerobic, and combined training all significantly reduced HbA1c, whereas significant fasting blood glucose reductions were restricted to resistance and combined training, both of which significantly outperformed aerobic training. Although cumulative ranking probabilities placed resistance training highest for eGFR, HbA1c, and fasting blood glucose, head-to-head active comparisons were largely non-significant and certainty of evidence was predominantly low, mandating that statistical hierarchies remain exploratory.

Crucially, primary sensitivity and subgroup evaluations provide essential caveats regarding effect stability. The prominent eGFR elevation observed with resistance training (+11.36 mL/min/1.73 m²) warrants cautious interpretation. In early diabetic nephropathy cohorts—which comprised 60% of eligible trials—acute filtration increases may partly reflect exercise-induced glomerular hyperfiltration or transient hemodynamic shifts rather than genuine parenchymal preservation (3). Furthermore, resistance training lost statistical significance for eGFR upon excluding high risk-of-bias trials, indicating that its apparent renal superiority was driven by low-quality studies. In contrast, combined training exhibited a more resilient metabolic profile, maintaining significant fasting blood glucose reductions across short- and long-term regimens and retaining moderate certainty of evidence under the CINeMA framework.

Collectively, the current evidence supports structured exercise as a beneficial non-pharmacological strategy in non-dialysis diabetic kidney disease, with multimodal regimens (such as combined training) offering balanced multi-target stability. Clinicians should nevertheless avoid rigid superiority assumptions and prioritize individualized exercise prescriptions tailored to patient functional capacity, baseline renal stage, and clinical priorities.

### Comparison with previous studies

Prior systematic reviews evaluating exercise therapy in renal populations have been substantially constrained by limited trial volumes, unstratified intervention modalities, or broad patient inclusion criteria. Nataraj et al. conducted a pairwise meta-analysis encompassing four RCTs (203 patients with diabetic nephropathy) and reported that exercise reduced UACR without significantly altering eGFR (6). Encompassing 20 RCTs and 1,746 patients specifically diagnosed with non-dialysis DKD, our network meta-analysis substantially expands the empirical baseline and refines these earlier observations. By resolving distinct exercise modalities within an interconnected network geometry, our primary findings indicate that renal functional adaptations are modality-dependent rather than uniform: RT yielded statistically significant improvements in eGFR over usual care and AT, whereas MBE selectively reduced UAE.

Our methodological framework also departs fundamentally from the meta-analysis by Cai and colleagues (5). Although Cai et al. aggregated 18 studies examining physical activity in diabetic nephropathy, their quantitative synthesis was predominantly informed by observational cohorts (14 non-randomized studies and only four RCTs) and collapsed disparate physical activities into a single generic exposure. In contrast, our review restricted eligibility strictly to randomized controlled trials and systematically categorized interventions into four distinct paradigms under the FITT framework. This design circumvents the confounding inherent in self-reported physical activity and demonstrates that therapeutic benefits in DKD are not a generic class effect of physical activity but are governed by the physiological mechanics of specific training modes.

Furthermore, while Liu et al. recently implemented a network meta-analysis of exercise modalities, their target cohort comprised general non-dialysis chronic kidney disease (7). Given that patients with DKD display unique metabolic disturbances—including severe peripheral insulin resistance, accelerated microvascular sclerosis, and abnormal intrarenal hemodynamics—findings derived from heterogeneous, non-diabetic CKD etiologies cannot be directly extrapolated to DKD clinical management. To our knowledge, the present study represents the first network meta-analysis specifically dedicated to evaluating distinct exercise modalities in an exclusive non-dialysis DKD population.

In addition to defining modality-specific hierarchies, this study implemented critical methodological refinements over previous reviews. First, unlike prior meta-analyses that conflated outcomes across disparate follow-up durations, we stratified interventions into short-term (≤12 weeks) and longer-term (>12 weeks) exposures. This temporal analysis revealed that while acute improvements in eGFR and UAE were predominantly observed within ≤12 weeks, glycemic benefits—specifically fasting blood glucose reductions mediated by RT and combined training (CT)—were robustly sustained across both short- and long-term regimens. Second, by incorporating the revised Cochrane RoB 2 tool and the CINeMA framework for the first time in this literature, we systematically appraised evidence certainty and uncovered important vulnerabilities. Notably, our primary sensitivity analysis demonstrated that the prominent eGFR benefit associated with RT was fragile and driven by high-risk trials, providing a calibrated, evidence-graded perspective that was absent in earlier syntheses.

Beyond the specific DKD population, our findings regarding the robust metabolic benefits of combined training align closely with the broader type 2 diabetes mellitus (T2DM) exercise literature (36). In the general T2DM population, multimodal regimens combining aerobic and resistance training have consistently demonstrated superior efficacy over isolated modalities not only in optimizing glycemic control, but also in improving physical functional capacity, cognitive performance, and overall health-related quality of life (37, 38). While these systemic benefits derived from non-nephropathy cohorts must be extrapolated to advanced DKD with caution, they strongly reinforce the clinical and physiological rationale for adopting combined training as a foundational, multi-target lifestyle intervention.

### Limitations

Several limitations of this systematic review and network meta-analysis warrant consideration. First, although the global network was interconnected, active head-to-head comparisons between distinct exercise modalities were sparse. Only three trials directly compared active exercise regimens (RT vs. AT, n=2; CT vs. AT, n=1), with the vast majority contrasting exercise against usual care. Consequently, relative comparative hierarchies among active modalities were predominantly informed by indirect statistical loops rather than direct head-to-head trial evidence, necessitating cautious interpretation of comparative rankings.

Second, the biological and clinical plausibility of specific effect magnitudes requires critical contextualization. In the primary analysis, resistance training elicited an eGFR improvement of 11.36 mL/min/1.73 m² (95% CI 4.47 to 18.25), an effect size that is disproportionately large relative to established nephroprotective pharmacotherapies and likely reflects bias-driven inflation from small-sample trials. This vulnerability is reinforced by our sensitivity analysis, in which statistical significance was abolished upon excluding high risk-of-bias studies (MD 8.24, 95% CI –1.17 to 17.64). Furthermore, because primary trials utilized disparate, non-standardized filtration equations (e.g., MDRD vs. CKD-EPI equations) rather than centralized biochemical recalibration, subtle measurement heterogeneity across cohorts was unavoidable. Concurrently, given that 60% of trials enrolled early diabetic nephropathy (Mogensen stage III), observed filtration increases in certain cohorts may partly mirror exercise-induced glomerular hyperfiltration or transient intrarenal hemodynamic shifts rather than sustained structural parenchymal preservation.

Third, transitivity may be nuanced by baseline disease spectrum and concurrent lifestyle interventions. Although quantitative transitivity assessments demonstrated acceptable balance in age and baseline glycemic levels (**Supplementary Table S8.1**), the network synthesized trials across CKD stages 1–4 and early diabetic nephropathy. Notably, trials evaluating mind–body exercise enrolled participants with substantially lower baseline HbA1c levels (mean 6.75%) than trials of other active modalities (means 8.2%–8.6%), indicating that mind–body cohorts exhibited a milder metabolic profile, which represents an inherent source of clinical transitivity variance. Additionally, several eligible trials incorporated non-exercise co-interventions, including traditional Chinese medicine, specialized nursing pathways, and structured dietary education. Although primary sensitivity analyses isolating pure exercise or retaining pragmatic nutritional support confirmed the broad stability of our primary conclusions, residual interactive confounding from lifestyle or supportive co-interventions cannot be definitively excluded.

Finally, severe reporting deficiencies regarding exercise dosing and safety monitoring constrained deeper exploratory syntheses. Inconsistencies in reporting exercise intensity, progression protocols, and adherence precluded meta-regression analyses based on the FITT framework. More critically, structured safety monitoring was documented in only five of the 20 included trials (25.0%), of which only three provided itemized event frequencies. This pronounced underreporting precluded quantitative network modeling of safety profiles, meaning that while reported events were non-serious and manageable, the universal clinical tolerability of high-intensity or multimodal exercise across the spectrum of non-dialysis DKD remains to be fully verified in adequately powered prospective trials.

### Implications for clinical practice

Contemporary clinical practice guidelines, including those from KDIGO (3), endorse regular physical activity as an essential lifestyle pillar in the comprehensive management of diabetic kidney disease. However, current recommendations remain predominantly generic, advising a cumulative weekly volume of moderate-intensity activity without granular specification regarding the comparative merits of distinct exercise paradigms (AT, RT, CT, or MBE). In the absence of definitive direct comparative trials, clinicians and exercise specialists often encounter uncertainty when formulating tailored regimens across varying disease stages and therapeutic priorities.

Given the exploratory nature of SUCRA rankings and the predominantly low certainty of evidence across comparisons, clinical recommendations must be framed prudently. Multimodal exercise combining aerobic and resistance training (CT) represents a balanced and pragmatic foundational strategy for patients with non-dialysis DKD. Although CT did not achieve statistical significance for eGFR elevation in the primary analysis, it conferred sustained, long-term glycemic benefits—maintaining statistically significant reductions in fasting blood glucose beyond 12 weeks—and was supported by moderate CINeMA evidence certainty. Conversely, while isolated RT yielded marked glycemic reductions and the largest nominal increase in eGFR, its apparent renal benefit was fragile upon sensitivity exclusion and may partly mirror early glomerular hyperfiltration. Therefore, clinical practice should avoid prematurely replacing multimodal regimens with isolated resistance training.

For patients with limited functional capacity, advanced age, or a perceived risk of exercise-induced musculoskeletal strain, mind–body exercise (MBE, such as Tai Chi and Baduanjin) represents a clinically attractive alternative or adjunct. In our network synthesis, MBE was the sole modality to elicit a statistically significant reduction in urinary albumin excretion compared with usual care. While the certainty of this evidence remains low and warrants further confirmation, the low-impact, highly accessible nature of MBE offers a feasible entry point to promote exercise adherence and address albuminuria in clinical practice. Additionally, exploratory analyses in the supplement indicated that mind–body exercise produced a significant reduction in systolic blood pressure (MD –15.70 mmHg; Supplementary Appendix 12), further reinforcing its clinically viable role in comprehensive hemodynamic and cardiovascular risk management in DKD.

At the operational level, individualized prescription and dynamic surveillance remain essential. Because systematic safety monitoring was documented in only 25% of eligible trials, exercise programs across the spectrum of non-dialysis DKD should initiate at low-to-moderate intensities and progress gradually under professional supervision. Dynamic monitoring of blood glucose is advisable to mitigate exercise-associated hypoglycemia, particularly during initial training phases. Rather than pursuing rigid algorithmic superiority rankings, clinicians should prioritize personalized exercise prescriptions that balance multi-target metabolic goals with individual tolerability, baseline renal stage, and long-term adherence.

### Implications for future research

Several critical avenues for future research emerge from the methodological and evidence gaps identified in this network meta-analysis. First, future clinical trials investigating exercise interventions in non-dialysis DKD must prioritize larger sample sizes, multi-center designs, and rigorous methodological adherence. To enable robust dose-response meta-regressions, future studies must adopt standardized and transparent reporting of exercise prescriptions, strictly implementing the FITT (Frequency, Intensity, Time, and Type) framework.

Second, given the chronic, progressive trajectory of non-dialysis DKD, the predominantly short follow-up periods in existing literature are insufficient to ascertain sustained long-term renal preservation. Rigorously designed, large-scale randomized controlled trials with extended follow-up durations are urgently needed to evaluate whether the physiological benefits of specific exercise modalities—such as the pronounced eGFR point estimates observed with resistance training—are durable over time. Furthermore, adverse event monitoring was documented in only 25% of current trials, future research must mandate rigorous, transparent safety reporting protocols to comprehensively establish the clinical tolerability of structured exercise across non-dialysis DKD.

Finally, future investigations should incorporate stratified trial designs across distinct non-dialysis CKD stages to elucidate potential stage-dependent variations in therapeutic responsiveness. Concurrently, there remains an empirical imperative for high-quality, direct head-to-head randomized trials comparing active exercise modalities, which will reduce reliance on indirect network evidence and generate definitive comparative hierarchies to guide precise clinical prescriptions.

## Conclusion

Structured exercise confers distinct, modality-dependent therapeutic benefits in patients with non-dialysis diabetic kidney disease. Combined aerobic and resistance training demonstrates favorable, duration-sustained reductions in fasting blood glucose with moderate evidence certainty, while mind–body exercise selectively reduces urinary albumin excretion. Although resistance training yielded the largest nominal improvements in filtration and glycemic metrics, its apparent renal benefit lacked stability against bias and warrants clinical caution. Importantly, because structured safety was formally monitored in only a minority of included trials, comparative clinical safety cannot be definitively concluded. Therefore, clinicians should avoid rigid superiority assumptions and prescribe tailored, multi-target exercise regimens aligned with patient functional capacity and disease priorities.

## Data Availability

The original contributions presented in the study are included in the article/**Supplementary Material**. Further inquiries can be directed to the corresponding author.
