# Supplementary material for: Comparative efficacy of different exercise modalities on renal function and glycemic control in patients with non-dialysis diabetic kidney disease: a systematic review and network meta-analysis
Source: Front Endocrinol (Lausanne). 2026 Oct 19;17:1991940. doi: 10.3389/fendo.2026.1991940 (PMC13724325; doi:10.3389/fendo.2026.1991940)
Supplement: Supplementary file 1 [file DataSheet1.pdf]

# Supplementary Appendix

## Comparative efficacy of different exercise modalities on renal function and glycemic control in patients with non-dialysis diabetic kidney disease: a systematic review and network meta-analysis

**Yihang Gong[1]，Jinhui Zheng[2]，Wenrui Huang[3]，Yongkyun Jeon***

**Table of contents**

Appendix 1: Search strategy ........ 2
Appendix 2: Characteristics of included studies ........ 8
Appendix 3: List of data extracted from the included randomized clinical trials ........ 13
Appendix 4: Risk of bias of randomized clinical trials ........ 14
Appendix 5: Evaluation of inconsistency and heterogeneity ........ 15
Appendix 6: SUCRA and cumulative probability plots ........ 17
Appendix7: League tables of relative treatment effects across exercise modalities ........ 21
Appendix 8: CINeMA Assessment ........ 25
Appendix 9: Funnel plots ........ 34
Appendix 10: Sensitivity analyses ........ 37
Appendix 11: Subgroup analysis ........ 41
Appendix 12: Exploratory Analyses ........ 42

## Appendix 1: Search strategy

**Table S1. Search strategy of PubMed**

| # | Searches |
|---|---|
| **1** | "Diabetic Nephropathies"[Mesh] |
| **2** | "diabetic nephropathy"[Title/Abstract] OR "diabetic nephropathies"[Title/Abstract] OR "diabetic kidney disease"[Title/Abstract] OR "diabetic kidney diseases"[Title/Abstract] OR "diabetic renal disease"[Title/Abstract] OR "DKD"[Title/Abstract] OR "DN"[Title/Abstract] |
| **3** | #1 OR #2 |
| **4** | "Exercise"[MeSH Terms] OR "Exercise Therapy"[MeSH Terms] OR "Physical Exertion"[MeSH Terms] OR "Resistance Training"[MeSH Terms] OR "High-Intensity Interval Training"[MeSH Terms] OR "Tai Ji"[MeSH Terms] OR "Yoga"[MeSH Terms] OR "Mind-Body Therapies"[MeSH Terms] OR "Walking"[MeSH Terms] OR "Running"[MeSH Terms] OR "Swimming"[MeSH Terms] |
| **5** | "exercise"[Title/Abstract] OR "exercises"[Title/Abstract] OR "exercising"[Title/Abstract] OR "physical activity"[Title/Abstract] OR "physical activities"[Title/Abstract] OR "physical exertion"[Title/Abstract] |
| **6** | "aerobic"[Title/Abstract] OR "resistance training"[Title/Abstract] OR "strength training"[Title/Abstract] OR "concurrent training"[Title/Abstract] OR "combined training"[Title/Abstract] OR "combined exercise"[Title/Abstract] OR "high intensity interval training"[Title/Abstract] OR "HIIT"[Title/Abstract] |
| **7** | "walking"[Title/Abstract] OR "running"[Title/Abstract] OR "jogging"[Title/Abstract] OR "swimming"[Title/Abstract] OR "cycling"[Title/Abstract] OR "bicycling"[Title/Abstract] OR "treadmill"[Title/Abstract] OR "dumbbell"[Title/Abstract] OR "resistance band"[Title/Abstract] OR "elastic band"[Title/Abstract] |
| **8** | "tai chi"[Title/Abstract] OR "taijiquan"[Title/Abstract] OR "yoga"[Title/Abstract] OR "qigong"[Title/Abstract] OR "baduanjin"[Title/Abstract] OR "liuzijue"[Title/Abstract] OR "mind-body"[Title/Abstract] OR "mind body"[Title/Abstract] |
| **9** | #4 OR #5 OR #6 OR #7 OR #8 |
| **10** | #3 AND #9 |
| **11** | "randomized controlled trial"[Publication Type] OR "controlled clinical trial"[Publication Type] OR "randomized"[Title/Abstract] OR "placebo"[Title/Abstract] OR "Clinical Trials as Topic"[MeSH Terms:noexp] OR "randomly"[Title/Abstract] OR "trial"[Title/Abstract] |
| **12** | #10 AND #11 |

**Table S2. Search strategy of Web of Science: Science Citation Index Expanded**

| # | Searches |
|---|---|
| **1** | TS=("diabetic nephropathy" OR "diabetic nephropathies" OR "diabetic kidney disease" OR "diabetic kidney diseases" OR "diabetic renal disease" OR "DKD" OR "DN") |
| **2** | TS=("exercise" OR "exercises" OR "exercising" OR "physical activity" OR "physical activities" OR "physical exertion" OR "exercise therapy") |
| **3** | TS=("aerobic" OR "resistance training" OR "strength training" OR "concurrent training" OR "combined training" OR "combined exercise" OR "high intensity interval training" OR "HIIT") |
| **4** | TS=("walking" OR "running" OR "jogging" OR "swimming" OR "cycling" OR "bicycling" OR "treadmill" OR "dumbbell" OR "resistance band" OR "elastic band") |
| **5** | TS=("tai chi" OR "taijiquan" OR "yoga" OR "qigong" OR "baduanjin" OR "liuzijue" OR "mind-body" OR "mind body" OR "mind-body therapies") |
| **6** | #2 OR #3 OR #4 OR #5 |
| **7** | #1 AND #6 |
| **8** | TS=("randomized controlled trial" OR "controlled clinical trial" OR "randomized" OR "placebo" OR "randomly" OR "trial" OR "RCT") |
| **9** | #7 AND #8 |

**Table S3. Search strategy of Embase**

| # | Searches |
|---|---|
| **1** | 'diabetic nephropathy'/exp OR 'diabetic kidney disease'/exp |
| **2** | 'diabetic nephropathy':ti,ab,kw OR 'diabetic nephropathies':ti,ab,kw OR 'diabetic kidney disease':ti,ab,kw OR 'diabetic kidney diseases':ti,ab,kw OR 'diabetic renal disease':ti,ab,kw OR 'dkd':ti,ab,kw OR 'dn':ti,ab,kw |
| **3** | #1 OR #2 |
| **4** | 'exercise'/exp OR 'kinesiotherapy'/exp OR 'physical activity'/exp OR 'resistance training'/exp OR 'high intensity interval training'/exp OR 'tai chi'/exp OR 'yoga'/exp OR 'mind body therapy'/exp OR 'walking'/exp OR 'running'/exp OR 'swimming'/exp |
| **5** | 'exercise':ti,ab,kw OR 'exercises':ti,ab,kw OR 'exercising':ti,ab,kw OR 'physical activity':ti,ab,kw OR 'physical activities':ti,ab,kw OR 'physical exertion':ti,ab,kw |
| **6** | 'aerobic':ti,ab,kw OR 'resistance training':ti,ab,kw OR 'strength training':ti,ab,kw OR 'concurrent training':ti,ab,kw OR 'combined training':ti,ab,kw OR 'combined exercise':ti,ab,kw OR 'high intensity interval training':ti,ab,kw OR 'hiit':ti,ab,kw |
| **7** | 'walking':ti,ab,kw OR 'running':ti,ab,kw OR 'jogging':ti,ab,kw OR 'swimming':ti,ab,kw OR 'cycling':ti,ab,kw OR 'bicycling':ti,ab,kw OR 'treadmill':ti,ab,kw OR 'dumbbell':ti,ab,kw OR 'resistance band':ti,ab,kw OR 'elastic band':ti,ab,kw |
| **8** | 'tai chi':ti,ab,kw OR 'taijiquan':ti,ab,kw OR 'yoga':ti,ab,kw OR 'qigong':ti,ab,kw OR 'baduanjin':ti,ab,kw OR 'liuzijue':ti,ab,kw OR 'mind-body':ti,ab,kw OR 'mind body':ti,ab,kw |
| **9** | #4 OR #5 OR #6 OR #7 OR #8 |
| **10** | #3 AND #9 |
| **11** | 'randomized controlled trial'/exp OR 'controlled clinical trial'/exp OR 'randomization'/exp OR 'double blind procedure'/exp OR 'single blind procedure'/exp OR 'placebo'/exp |
| **12** | 'randomized':ti,ab,kw OR 'placebo':ti,ab,kw OR 'randomly':ti,ab,kw OR 'trial':ti,ab,kw OR 'rct':ti,ab,kw |
| **13** | #11 OR #12 |
| **14** | #10 AND #13 |

**Table S4. Search strategy of Cochrane Central Register of Controlled Trials (CENTRAL)**

| # | Searches |
|---|---|
| **1** | MeSH descriptor: [Diabetic Nephropathies] explode all trees |
| **2** | ("diabetic nephropathy" OR "diabetic nephropathies" OR "diabetic kidney disease" OR "diabetic kidney diseases" OR "diabetic renal disease" OR "DKD" OR "DN"):ti,ab,kw |
| **3** | #1 OR #2 |
| **4** | MeSH descriptor: [Exercise] explode all trees OR MeSH descriptor: [Exercise Therapy] explode all trees OR MeSH descriptor: [Physical Exertion] explode all trees OR MeSH descriptor: [Resistance Training] explode all trees OR MeSH descriptor: [High-Intensity Interval Training] explode all trees OR MeSH descriptor: [Tai Ji] explode all trees OR MeSH descriptor: [Yoga] explode all trees OR MeSH descriptor: [Mind-Body Therapies] explode all trees |
| **5** | ("exercise" OR "exercises" OR "exercising" OR "physical activity" OR "physical activities" OR "physical exertion"):ti,ab,kw |
| **6** | ("aerobic" OR "resistance training" OR "strength training" OR "concurrent training" OR "combined training" OR "combined exercise" OR "high intensity interval training" OR "HIIT"):ti,ab,kw |
| **7** | ("walking" OR "running" OR "jogging" OR "swimming" OR "cycling" OR "bicycling" OR "treadmill" OR "dumbbell" OR "resistance band" OR "elastic band"):ti,ab,kw |
| **8** | ("tai chi" OR "taijiquan" OR "yoga" OR "qigong" OR "baduanjin" OR "liuzijue" OR "mind-body" OR "mind body"):ti,ab,kw |
| **9** | #4 OR #5 OR #6 OR #7 OR #8 |
| **10** | #3 AND #9 |
| **11** | MeSH descriptor: [Randomized Controlled Trials as Topic] explode all trees OR MeSH descriptor: [Controlled Clinical Trials as Topic] explode all trees OR ("randomized" OR "placebo" OR "randomly" OR "trial" OR "RCT"):ti,ab,kw |
| **12** | #10 AND #11 |

**Table S5. Search strategy of CNKI (China National Knowledge Infrastructure)**

| # | Searches |
|---|---|
| **1** | SU=("糖尿病肾病" OR "糖尿病肾脏疾病" OR "糖尿病性肾病" OR "DKD" OR "DN") |
| **2** | SU=("运动" OR "锻炼" OR "体力活动" OR "身体活动" OR "运动疗法" OR "运动干预") |
| **3** | SU=("有氧" OR "抗阻" OR "力量训练" OR "高强度间歇" OR "HIIT" OR "联合训练" OR "联合运动" OR "并行训练") |
| **4** | SU=("步行" OR "散步" OR "快走" OR "跑步" OR "慢跑" OR "游泳" OR "骑行" OR "自行车" OR "跑步机" OR "哑铃" OR "弹力带" OR "阻力带") |
| **5** | SU=("太极" OR "太极拳" OR "八段锦" OR "六字诀" OR "气功" OR "瑜伽" OR "身心运动" OR "传统功法") |
| **6** | #2 OR #3 OR #4 OR #5 |
| **7** | #1 AND #6 |
| **8** | SU=("随机" OR "随机对照" OR "随机分组" OR "随机分配" OR "临床试验" OR "RCT") |
| **9** | #7 AND #8 |

**Table S6. Search strategy of Wanfang Data**

| # | Searches |
|---|---|
| **1** | 主题:("糖尿病肾病" OR "糖尿病肾脏疾病" OR "糖尿病性肾病" OR "DKD" OR "DN") |
| **2** | 主题:("运动" OR "锻炼" OR "体力活动" OR "身体活动" OR "运动疗法" OR "运动干预") |
| **3** | 主题:("有氧" OR "抗阻" OR "力量训练" OR "高强度间歇" OR "HIIT" OR "联合训练" OR "联合运动" OR "并行训练") |
| **4** | 主题:("步行" OR "散步" OR "快走" OR "跑步" OR "慢跑" OR "游泳" OR "骑行" OR "自行车" OR "跑步机" OR "哑铃" OR "弹力带" OR "阻力带") |
| **5** | 主题:("太极" OR "太极拳" OR "八段锦" OR "六字诀" OR "气功" OR "瑜伽" OR "身心运动" OR "传统功法") |
| **6** | #2 OR #3 OR #4 OR #5 |
| **7** | #1 AND #6 |
| **8** | 主题:("随机" OR "随机对照" OR "随机分组" OR "随机分配" OR "临床试验" OR "RCT") |
| **9** | #7 AND #8 |

**Appendix 2: Characteristics of included studies**

**Table S2.1:** Baseline of characteristics of included studies

| Study | Country | Sample size(I/C) | Age,years (mean±SD)(I/C) | Male (%) (I/C) | Diabetes duration, years(mean±SD) (I/C) | Clinical Stage | BMI(kg/m²)(mean±SD) (I/C) | HbA1c（%）(mean±SD) (I/C) | Intervention | Exercise program | Frequency (sessions/week) | Intensity | Session duration (min) | Duration (weeks) | Comparator |
|---|---|---|---|---|---|---|---|---|---|---|---|---|---|---|---|
| Leehey 2016 | USA | 18/18 | 65.4 ± 8.7 / 66.6 ± 7.5 | 100% / 100% | NR | CKD 2-4 | 36.2 ± 4.8 / 37.4 ± 4.2 | 7.8 ± 2.1 / 7.9 ± 1.5 | CT | Treadmill, Elliptical, Weights | 3 | 25%~84% $VO_2$peak | 80–90 | 52 | UC |
| Leehey 2009 | USA | 7/6 | 66 (median, range 55-81) | 100% / 100% | NR | CKD 2-4 | 34.15 ± 3.39 / 33.82 ± 3.47 | 7.4 ± 1.1 / 8.2 ± 1.7 | AT | Treadmill walking | 3 | 25-44%→60-84% $VO_2$peak | 30–40 | 24 | UC |
| Nataraj 2024 | India | 30/30 | 59.0 ± 7.60 / 58.3 ± 7.78 | 86.7%/ 86.7% | 11 (mean only) / 11 (mean only) | CKD 1-4 | 26.2 ± 4.65 / 26.2 ± 3.63 | NR | CT | Walking, Body-weight exercises | AT3-5；RT2-3 | Borg RPE(11-13） | 40–55 | 24 | UC |
| Waki 2024 | Japan | 66/60 | 58.6 ± 10.1 / 60.3 ± 8.7 | 71.2%/ 76.7% | 14.0 ± 7.9 / 12.6 ± 6.4 | CKD 1-3 | 28.7 ± 5.3 / 28.3 ± 4.0 | 7.5 ± 0.81 / 7.5 ± 0.74 | AT | Smartphone-guided walking | 7 | Maximum heart rate 60%-70% | 150 min/week | 48 | UC |
| Hadjadj 2026 | France/ Belgium | 59/44 | 66.2 ± 8.5 / 65.9 ± 6.7 | 76% / 66% | 18.0(median, IQR) / 17.0 (median, IQR) | CKD 1-3 | 33.0 ± 7.6 / 32.8 ± 7.1 | 7.4 ± 1.11 / 7.2 ± 1.04 | CT | Strengthening + Intermittent HIPA | 2 | High-intensity interval | NR | 104 | UC |
| Dong 2019 | China | 60/61 | 56 ± 14.1 (overall) | 59.5%/ 59.5% | 7-12(range)/ 7-12 (range) | CKD 2-3 | 28.6±1.8 /28±1.4 | 8.74 ± 0.78/ 8.49 ± 0.26 | AT | Brisk walking | 7 | Moderate intensity | 45–60 | 24 | UC |
| Moghadasi 2022 | Iran | 15/15 | 52.8 ± 7.3 (overall) | 100% / 100% | >5 (range only) / >5 (range only) | CKD 2-4 | 27.3±4.2/ 27.8±2.3 | NR | RT | 6-exercise circuit machines | 3 | 50-65% 1RM→65-80% 1RM | 60 | 8 | UC |
| Li 2024* | China | 30/30 | 36.60±13.16/ 37.01± 13.02 | 44% / 44.8% | ≥1 (range only) / ≥1 (range only) | EDN | 29.98±5.77 / 29.93±6.01 | 8.42 ± 1.51/ 8.39 ± 1.52 | RT | Resistance band / Running | 5 | 60-80% HRmax | 40 | 12 | AT |

| | | | | | | | | | | | | | | | |
|---|---|---|---|---|---|---|---|---|---|---|---|---|---|---|---|
| Lin 2012 | China | 30/30 | 60 ± 2.2 / 60 ± 2.2 | 51.1%/ 51.1% | 2-10 (range) /2-10(range) | EDN | NR | NR | MBE | 24-style Tai Chi, Tai Chi stance | 7 | Low intensity | 90 | 12 | UC |
| Liu 2020 | China | 38/37 | 62.2 ± 3.8 / 63.1 ± 4.2 | 55.3%/ 54.1% | 12.52 ± 3.12 /12.78± 3.28 | EDN | 27.12±5.36 / 27.36±5.29 | 8.36 ± 1.22 /8.41 ± 1.15 | CT | Resistance (squats, weights) / Aerobics | RT：3-4；AT：3-5 | RT：70-80% 1RM；AT：Low intensity | 20–30 | 12 | UC |
| Cui 2026 | China | 46/47 | NR | 54.3%/ 51.1% | NR | EDN | NR | 8.72 ± 1.22 /8.94 ± 1.04 | AT | Walking, Running, Gymnastics, Tai Chi | ≥3 | medium-low intensity | 30 | 12 | UC |
| Sun 2018 | China | 33/33 | 50.6 ± 8.2 / 51.8 ± 9.8 | 48.5%/ 45.5% | NR | EDN | NR | 8.78 ± 1.35 / 8.74 ± 1.4 | MBE | Liuzijue-Qigong | 14 | Low intensity | 30 | 12 | UC |
| Li 2025 | China | 90/90 | 58.27±11.89/ 57.35± 11.36 | 58.9%/ 56.7% | 8.13 ± 2.53 / 7.79 ± 2.65 | EDN | NR | 8.71 ± 0.6 / 8.76 ± 0.58 | CT | Resistance machines, walking/cycling | RT 2-3 AT 3-5 | RT 8~12 RM,AT50%~70%$VO_2$peak | 30–60 | 8 | UC |
| Kong 2017* | China | 30/30 | 48.3 ± 10.7 / 49.1 ± 9.3 | 56.7%/ 53.3% | 7.4 ± 6.9 / 7.6 ± 6.5 | EDN | NR | 9.2 ± 2.2 / 9.3 ± 2 | RT | Elastic band / Running | 3 | Tolerable | 30 | 24 | AT |
| Tong 2026 | China | 52/52 | 69.90±4.31/ 70.05± 4.62 | 53.8%/ 51.9% | 12.33 ± 3.09 /12.42± 3.58 | CKD 2-3 | 23.10±1.32 / 23.16±1.77 | 8.65 ± 1.15 /8.76 ± 1.44 | CT | Dumbbells, band squats, cycling/running | ≥3 | Moderate | 20–30 | 12 | UC |
| Zhong 2023* | China | 52/51 | 54.12±7.79/ 53.74± 7.85 | 63.5%/ 68.6% | 9.82 ± 2.43 / 9.73 ± 2.31 | EDN | 25.12±1.31 / 24.12±1.13 | 8.91 ± 0.75/ 8.86 ± 0.69 | CT | Aerobic gymnastics, band squats | AT：3；RT：3-4 | Tolerable | 50 (20+30) | 8 | AT |
| Tian 2026 | China | 130/130 | 63.04±8.21/ 62.87± 7.01 | 58.5%/ 56.2% | 10.08 ± 2.37 / 9.94 ± 2.25 | EDN | 23.47±3.62 / 23.52±3.72 | 6.25 ± 1 / 6.23 ± 1.02 | MBE | Baduanjin | 7 | Low intensity | 30 | 24 | UC |

| | | | | | | | | | | | | | | | |
|---|---|---|---|---|---|---|---|---|---|---|---|---|---|---|---|
| Wu 2024 | China | 30/30 | 55.03±8.86/ 58.13± 9.35 | NR | NR | EDN | NR | 10.54± 3.93 /11.09±4.38 | CT | Dumbbell gymnastics (11 sections) | 5 | Moderate-low | 30 | 24 | UC |
| Wu 2025 | China | 25/25 | 67.04±7.52/ 66.03± 8.48 | 64.0%/ 56.0% | 11.54± 3.27/ 10.97 ± 3.05 | EDN | NR | 8.21 ± 1.3 / 8.16 ± 1.21 | AT | Walk, run, swim, cycling | 5 | 50-70% of maximum heart rate | 30–40 | 4 | UC |
| Fang 2025 | China | 43/43 | 60.88±3.64/ 59.78± 3.80 | 58.1%/ 60.5% | 4.54 ± 1.32 / 4.84 ± 1.22 | EDN | NR | 8.2 ± 1.34 / 8.15 ± 1.25 | RT | Grip ball, Compression band | 7 | Grip strength > 20 kPa； | 15–20 | 12 | UC |

Note: Baseline continuous variables are expressed as mean ± standard deviation (SD), median (interquartile range [IQR] or range), or percentage (%). Exercise prescriptions are detailed in accordance with the FITT framework (Frequency, Intensity, Time [session duration], and Type [exercise program]).

Abbreviations: 1RM, 1-repetition maximum; AT, aerobic training; BMI, body mass index; Borg RPE, Borg rating of perceived exertion; CKD, chronic kidney disease; CT, combined training (aerobic plus resistance); EDN, early diabetic nephropathy; FITT, frequency, intensity, time, and type; HbA1c, glycated hemoglobin; HIPA, high-intensity physical activity; HRmax, maximum heart rate; I/C, intervention group / comparator group; IQR, interquartile range; MBE, mind–body exercise; NR, not reported; RT, resistance training; SD, standard deviation; UC, usual care; $VO_2$peak, peak oxygen consumption.

* Head-to-head comparison trial between active exercise modalities.

**Table S2.2: Definition of exercises**

| Exercise category | Exercise category definition | Specific exercise type | Description of specific exercise type |
|---|---|---|---|
| Aerobic Training | Aerobic training refers to the type of repetitive, structured physical activity that requires the body's aerobic metabolic system to use oxygen to produce energy for sustained periods. | Walking | Walking involves forward locomotion by alternate stepping of the feet, typically performed at low-to-moderate intensity on flat or inclined surfaces, with variations including treadmill, Nordic, or backward walking. |
| | | Swimming | Swimming is an in-water exercise that utilizes the body's natural buoyancy combined with coordinated arm and leg movements to propel the body through water. |
| | | Cycling | Cycling involves pedaling on a stationary or outdoor bicycle, primarily engaging the lower extremity and core musculature. |
| Resistance Training | Resistance training (also called strength training) refers to active exercise done by overcoming resistance; it's when muscles actively work against external resistance. | Equipment- based resistance training | This involves using dumbbells, barbells, resistance bands, or other external loads to perform strength exercises. |
| | | Bodyweight training | This uses one's own body weight as resistance, including exercises such as push- ups, pull- ups, and squats, and is suitable for beginners or settings without equipment. |
| | | Progressive overload | This refers to gradually increasing training intensity (e.g., weight, sets, repetitions) to continuously stimulate muscle adaptation and growth. |
| | | Isometric training | This involves maintaining muscle tension through static contractions without joint movement, which is suitable for beginners and can effectively enhance muscle strength. |
| Concurrent Training | Concurrent training incorporates both aerobic training and resistance training. | | |
| Mind–Body Exercise | Mind–body exercise is a form of physical activity that combines controlled movements, full-body stretching, breathing techniques, and a meditation component。 | Tai Chi | Tai Chi is a traditional Chinese mind–body exercise that combines slow, flowing physical movements with meditation and breathing control. |
| | | Baduanjin | Baduanjin is a traditional Chinese health Qigong routine consisting of eight simple movements, performed as a mind-body focused exercise. |
| | | Yoga | Yoga is a mind–body practice that originated in ancient India, combining physical postures, breathing techniques, and meditation. |
| | | Qigong | Qigong is a traditional Chinese |

| Exercise category | Exercise category definition | Specific exercise type | Description of specific exercise type |
|---|---|---|---|
| | | | mind–body practice that integrates gentle rhythmic movements, breathing techniques, and meditation to cultivate and balance vital energy. |
| control group | The control group serves as the baseline for comparison to assess the impact of the experimental intervention. | Usual Care | Usual care refers to the standard or routine clinical care that patients would normally receive in practice, without additional protocolized study interventions. |
| | | Health Education | Health education refers to basic health information provided to participants in the study, rather than experimental health education or treatment interventions. |

**Appendix 3: List of data extracted from the included randomized clinical trials**

| Data category | List of variables |
|---|---|
| Study | Primary author, year of publication, country, study design, and sample size across groups |
| Patients | Age, sex distribution (% male), diabetes duration, baseline BMI, baseline HbA1c (%), and clinical CKD stage / EDN classification. |
| Interventions | Specific exercise modality(AT, RT, CT, MBE) , exercise activities, frequency (sessions/week), intensity, session duration (min), total intervention duration (weeks), and comparator details. |
| Efficacy outcome | Estimated glomerular filtration rate (eGFR, mL/min/1.73 m²), urinary albumin excretion (UAE, assessed via UACR [mg/g] or 24-h UAER [mg/24h]), glycated hemoglobin (HbA1c, %), and fasting blood glucose (FBG, mmol/L). |
| Adverse events | Various adverse events reported in included trials |

**Abbreviations:** AT, aerobic training; BMI, body mass index; CKD, chronic kidney disease; CT, combined training; EDN, early diabetic nephropathy; eGFR, estimated glomerular filtration rate; FBG, fasting blood glucose; HbA1c, glycated hemoglobin; MBE, mind–body exercise; RT, resistance training; UACR, urinary albumin-to-creatinine ratio; UAE, urinary albumin excretion; UAER, urinary albumin excretion rate.

**Appendix 4: Risk of bias of randomized clinical trials**

**Table S4: Study level risk of bias assessment using Cochrane risk of bias tool 2.0 for assessing risk of bias of randomized clinical trials.**

| Unique ID | Randomization process | Deviations from intended interventions | Missing outcome data | Measurement of the outcome | Selection of the reported result | Overall |
|---|---|---|---|---|---|---|
| Leehey 2016 | Low | Some concerns | High | Low | Low | High |
| Leehey 2009 | Low | Low | Some concerns | Low | Some concerns | Some concerns |
| Nataraj 2024 | Low | Low | Low | Low | Low | Low |
| Waki 2024 | Low | Low | Low | Low | Low | Low |
| Hadjadj 2026 | Low | Some concerns | Some concerns | Low | Low | Some concerns |
| Dong 2019 | Low | Some concerns | Low | Low | Low | Some concerns |
| Moghadasi 2022 | Low | Some concerns | High | Low | Low | High |
| Li 2024 | Low | Some concerns | Low | Low | Low | Some concerns |
| Lin 2012 | Low | Low | Low | Low | Low | Low |
| Liu 2020 | Low | Some concerns | Low | Low | Low | Some concerns |
| Cui 2026 | Low | Low | Some concerns | Low | Low | Some concerns |
| Sun 2018 | Low | Low | Low | Low | Low | Low |
| Li 2025 | Low | Low | Low | Low | Low | Low |
| Kong 2017 | Low | Low | Low | Low | Low | Low |
| Tong 2026 | Low | Low | Low | Low | Low | Low |
| Zhong 2023 | Low | Low | Low | Low | Low | Low |
| Tian 2026 | Low | Some concerns | Low | Low | Low | Some concerns |
| Wu 2024 | Low | Low | Low | Low | Low | Low |
| Wu 2025 | Low | Some concerns | Low | Low | Low | Some concerns |
| Fang 2025 | Low | Low | Low | Low | Low | Low |

**Appendix 5: Evaluation of inconsistency and heterogeneity**

**Table S5.1:** Global consistency

| Clinical outcome | Chi square | P value | $\tau^2$ network |
|---|---|---|---|
| eGFR | 1.45 | 0.4831 | 13.12 |
| UAE(UACR or UAER) | 2.18 | 0.1401 | 0.73 |
| HbA1c | 1.92 | 0.3834 | 0.55 |
| FBG | 8.12 | 0.0673 | 0.69 |

Abbreviations: FBG, fasting blood glucose; HbA1c, hemoglobin A1c; UAE, urinary albumin excretion, assessed by UACR (mg/g) or UAER (mg/24h); eGFR, estimated glomerular filtration rate.

**Table S5.2:** Node-splitting of eGFR. Testing the inconsistency between direct and indirect comparisons in different exercise interventions

| Side | Direct | | Indirect | | Difference | | | |
|---|---|---|---|---|---|---|---|---|
| | Coef. | Std.Err. | Coef. | Std.Err. | Coef. | Std.Err. | P>\|z\| | tau |
| ATv.s.CT | 0.2 | 6.068951 | 1.59095 | 3.40573 | -1.39095 | 6.95925 | 0.842 | 4.126577 |
| ATv.s.RT | 6.009363 | 3.67705 | 14.54168 | 5.56782 | -8.532312 | 6.685544 | 0.202 | 3.14942 |
| ATv.s.UC | -1.683866 | 2.414964 | -6.784237 | 4.597661 | 5.100371 | 5.162117 | 0.323 | 3.734704 |
| CTv.s.UC | -4.329783 | 2.560363 | -2.92447 | 6.547402 | -1.405312 | 6.957292 | 0.84 | 4.127629 |
| RTv.s.UC | -16.39132 | 5.177102 | -7.824999 | 4.233279 | -8.566318 | 6.688113 | 0.2 | 3.148 |

Abbreviations: eGFR, estimated glomerular filtration rate;AT, aerobic training; RT, resistance training; CT, combined training (aerobic plus resistance training); UC, usual care.

**Table S5.3:** Node-splitting of UAE(UACR or UAER). Testing the inconsistency between direct and indirect comparisons in different exercise interventions.

| Side | Direct | | Indirect | | Difference | | | |
|---|---|---|---|---|---|---|---|---|
| | Coef. | Std.Err. | Coef. | Std.Err. | Coef. | Std.Err. | P>\|z\| | tau |
| ATv.s.CT | -0.8030719 | 0.8157948 | 0.730279 | 0.6438088 | -1.533351 | 1.039236 | 0.14 | 0.7895781 |
| ATv.s.UC | 1.028918 | 0.4285291 | -0.5039581 | 0.9468305 | 1.532877 | 1.039067 | 0.14 | 0.789557 |
| CTv.s.UC | 0.2985836 | 0.4809956 | 1.832 | 0.9214895 | -1.533416 | 1.039242 | 0.14 | 0.7895783 |
| MBEv.s.UC | 1.414044 | 0.638627 | 0.1173018 | 141.448 | 1.296742 | 141.4498 | 0.993 | 0.8561 |

Abbreviations: UAE, urinary albumin excretion, assessed by UACR (mg/g) or UAER (mg/24h);AT, aerobic training; RT, resistance training; CT, combined training (aerobic plus resistance training); MBE, mind-body exercise; UC, usual care.

**Table S5.4:** Node-splitting of HbA1c. Testing the inconsistency between direct and indirect comparisons in different exercise interventions.

| Side | Direct | | Indirect | | Difference | | | |
|---|---|---|---|---|---|---|---|---|
| | Coef. | Std.Err. | Coef. | Std.Err. | Coef. | Std.Err. | P>\|z\| | tau |
| AT v.s.CT | -0.44 | 0.7176943 | 0.7226095 | 0.4748195 | -1.16261 | 0.8605455 | 0.177 | 0.7056263 |
| AT v.s.RT | -0.6751585 | 0.6332449 | -0.3907228 | 0.8875887 | -0.2844356 | 1.090233 | 0.794 | 0.7740223 |
| AT v.s.UC | 1.324587 | 0.3650766 | 0.390262 | 0.6071525 | 0.9343253 | 0.7086183 | 0.187 | 0.7044137 |
| CT v.s.UC | 0.5369453 | 0.3308739 | 1.699651 | 0.7948035 | -1.162705 | 0.860541 | 0.177 | 0.7056236 |
| MBE v.s.UC | 0.376551 | 0.5539943 | 1.769645 | 141.4539 | -1.393094 | 141.4552 | 0.992 | 0.7411585 |
| RT v.s.UC | 1.489998 | 0.8112164 | 1.774409 | 0.7283951 | -0.2844108 | 1.090244 | 0.794 | 0.7740231 |

Abbreviations: HbA1c, hemoglobin A1c;AT, aerobic training; RT, resistance training; CT, combined training (aerobic plus resistance training); MBE, mind-body exercise; UC, usual care.

**Table S5.5:** Node-splitting of FBG. Testing the inconsistency between direct and indirect comparisons in different exercise interventions.

| Side | Direct | | Indirect | | Difference | | | |
|---|---|---|---|---|---|---|---|---|
| | Coef. | Std.Err. | Coef. | Std.Err. | Coef. | Std.Err. | P>\|z\| | tau |
| AT v.s.CT | -1.7 | 0.9785153 | -1.910172 | 0.7361708 | 0.2101723 | 1.224516 | 0.864 | 0.9382344 |
| AT v.s.RT | -3.58919 | 0.8331194 | -0.573454 | 0.6171942 | -3.015736 | 1.039042 | 0.063 | 0.5806336 |
| AT v.s.UC | 0.4196668 | 0.4932694 | -1.112706 | 0.7446595 | 1.532373 | 0.8920342 | 0.086 | 0.7808166 |
| CT v.s.UC | 1.745772 | 0.4992447 | 1.53504 | 1.116314 | 0.2107319 | 1.224592 | 0.863 | 0.9382535 |
| MBE v.s.UC | 0.6905271 | 0.6534714 | -0.8759916 | 141.4104 | 1.566519 | 141.4126 | 0.991 | 0.8316424 |
| RT v.s.UC | 0.9076796 | 0.5140646 | 3.923282 | 0.9004239 | -3.015602 | 1.039042 | 0.054 | 0.5806338 |

Abbreviations: FBG, fasting blood glucose;AT, aerobic training; RT, resistance training; CT, combined training (aerobic plus resistance training); MBE, mind-body exercise; UC, usual care

**Appendix 6: SUCRA and cumulative probability plots**

**Figure S6.1:** Cumulative ranking curve plots of different interventions for eGFR.

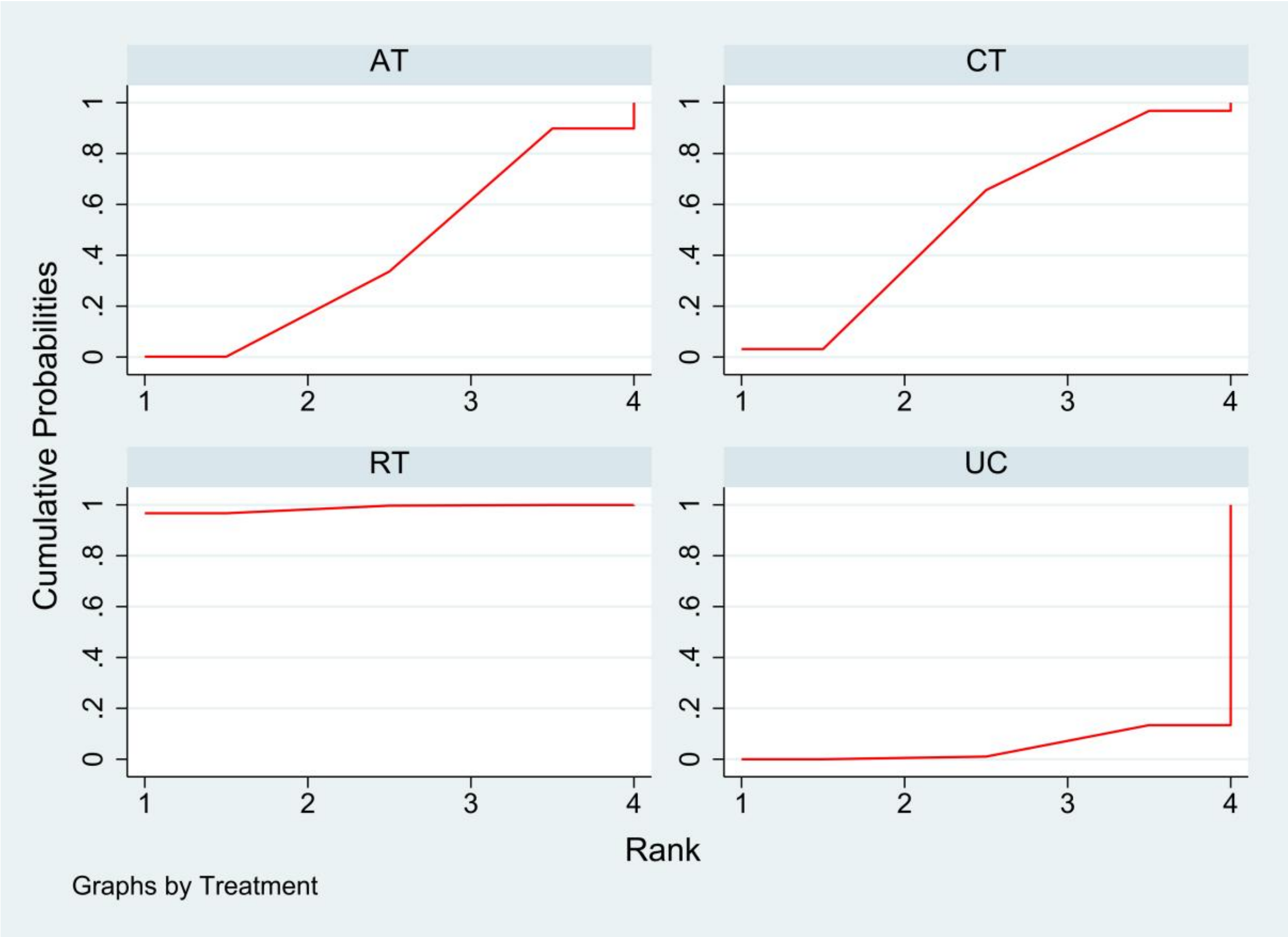


Abbreviations: eGFR, estimated glomerular filtration rate;AT, aerobic training; RT, resistance training; CT, combined training (aerobic plus resistance training); UC, usual care.

**Table S6.1:** SUCRA values of different interventions for eGFR.

| Treatment | SUCRA | PrBest | MeanRank |
|---|---|---|---|
| AT | 41. 2 | 0. 2 | 2. 8 |
| CT | 55. 2 | 3. 1 | 2. 3 |
| RT | 98. 8 | 96. 7 | 1 |
| UC | 4. 8 | 0 | 3. 9 |

Abbreviations: SUCRA, surface under the cumulative ranking curve.

**Figure S6.2:**Cumulative ranking curve plots of different interventions for UAE(UACR or UAER).

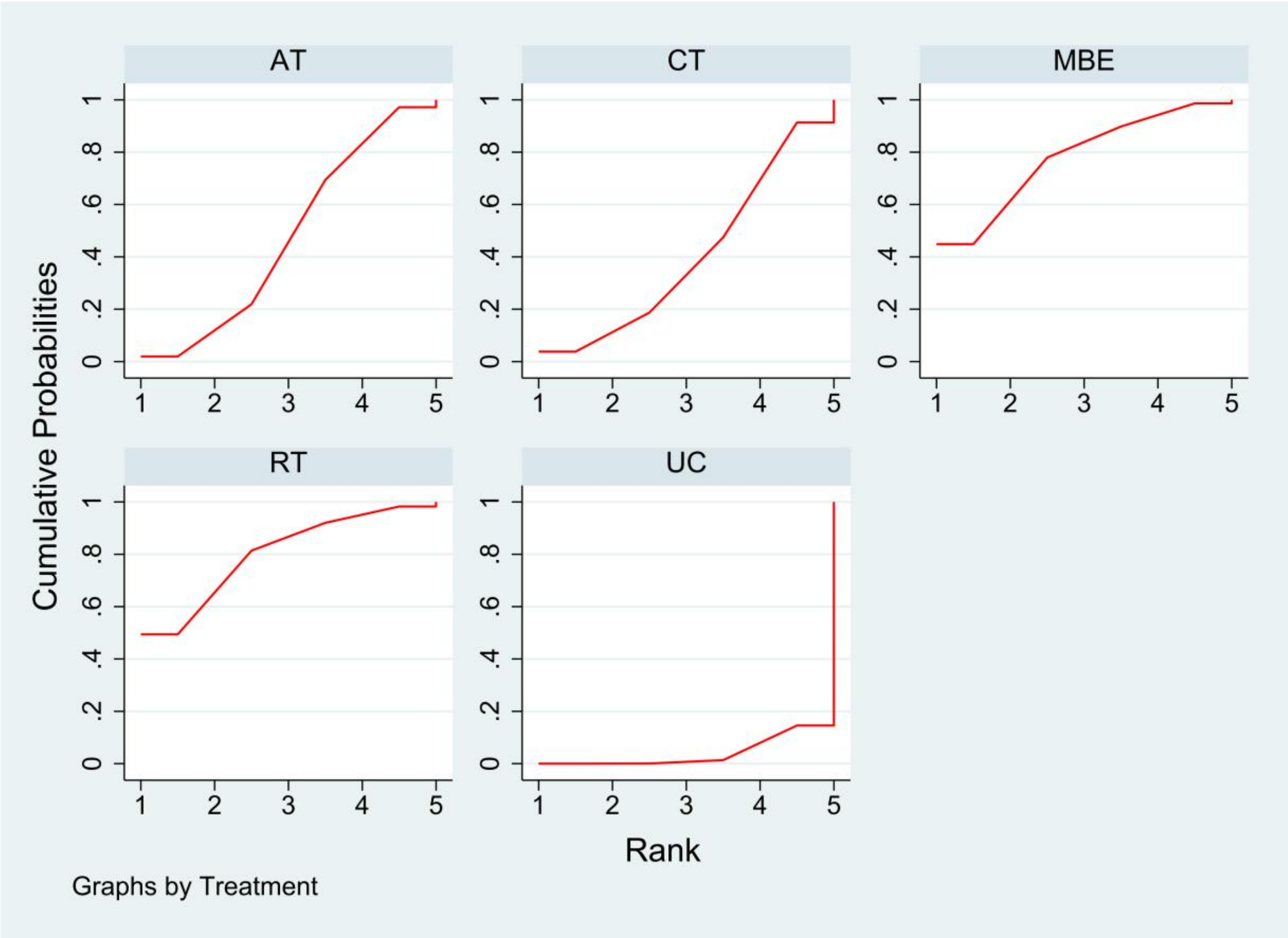


Abbreviations: UAE, urinary albumin excretion, assessed by UACR (mg/g) or UAER (mg/24h), urinary albumin excretion rate;AT, aerobic training; RT, resistance training; CT, combined training (aerobic plus resistance training); MBE, mind-body exercise; UC, usual care.

**Table S6.2:** SUCRA values of different interventions UAE(UACR or UAER).

| Treatment | SUCRA | PrBest | MeanRank |
|---|---|---|---|
| AT | 47. 6 | 1. 9 | 3. 1 |
| CT | 40. 3 | 3. 8 | 3. 4 |
| MBE | 77. 8 | 44. 9 | 1. 9 |
| RT | 80. 3 | 49. 4 | 1. 8 |
| UC | 4 | 0 | 4. 8 |

Abbreviations: SUCRA, surface under the cumulative ranking curve.

**Figure S6.3:**Cumulative ranking curve plots of different interventions for HbA1c.

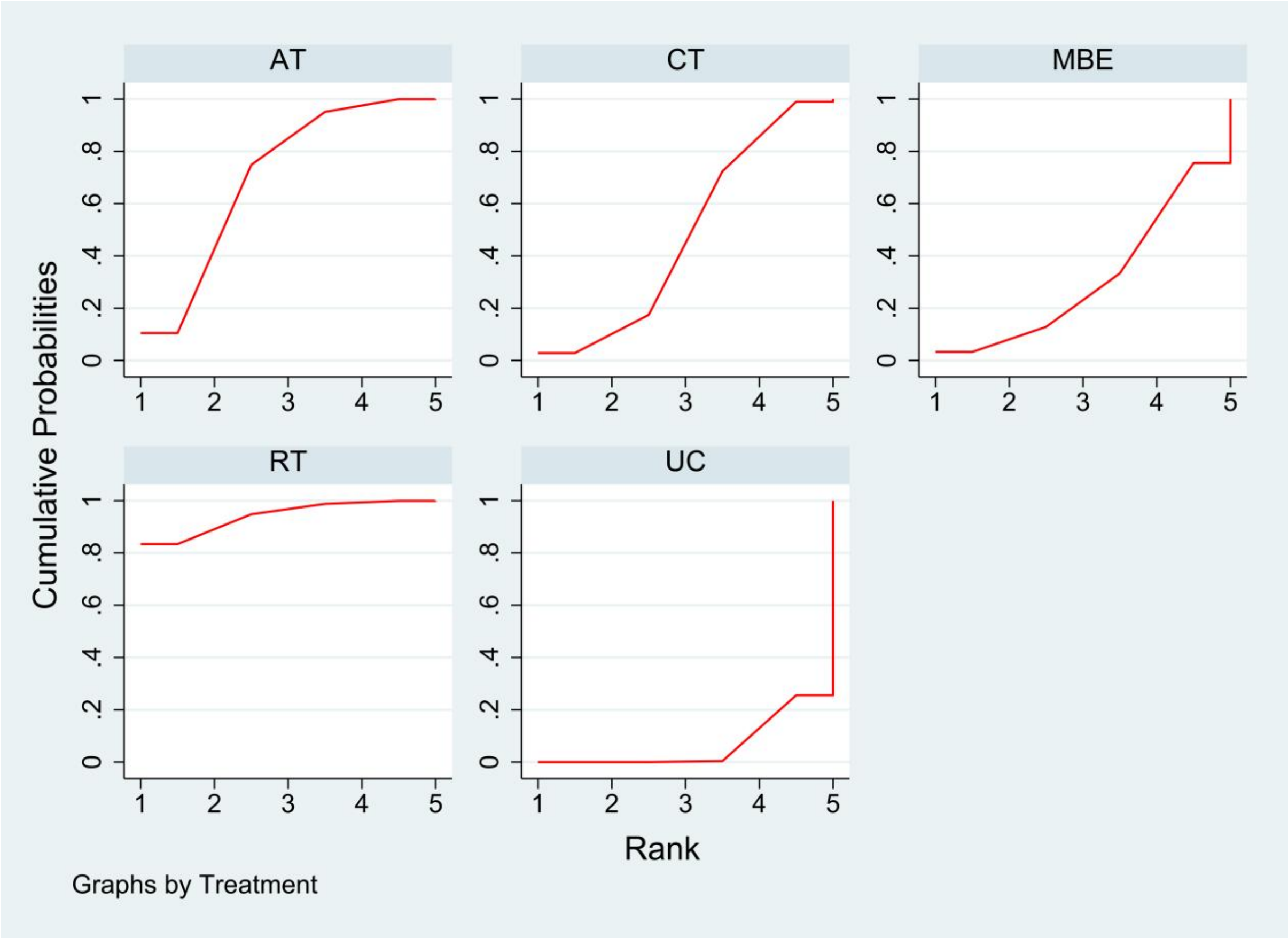


Abbreviations: HbA1c, hemoglobin A1c;AT, aerobic training; RT, resistance training; CT, combined training (aerobic plus resistance training); MBE, mind-body exercise; UC, usual care.

**Table S6.3:** SUCRA values of different interventions for HbA1c.

| Treatment | SUCRA | PrBest | MeanRank |
|---|---|---|---|
| AT | 70. 1 | 10. 5 | 2. 2 |
| CT | 47. 9 | 2. 8 | 3. 1 |
| MBE | 31. 3 | 3. 3 | 3. 7 |
| RT | 94. 2 | 83. 4 | 1. 2 |
| UC | 6. 5 | 0 | 4. 7 |

Abbreviations: SUCRA, surface under the cumulative ranking curve.

**Figure S6.4:**Cumulative ranking curve plots of different interventions for FBG.

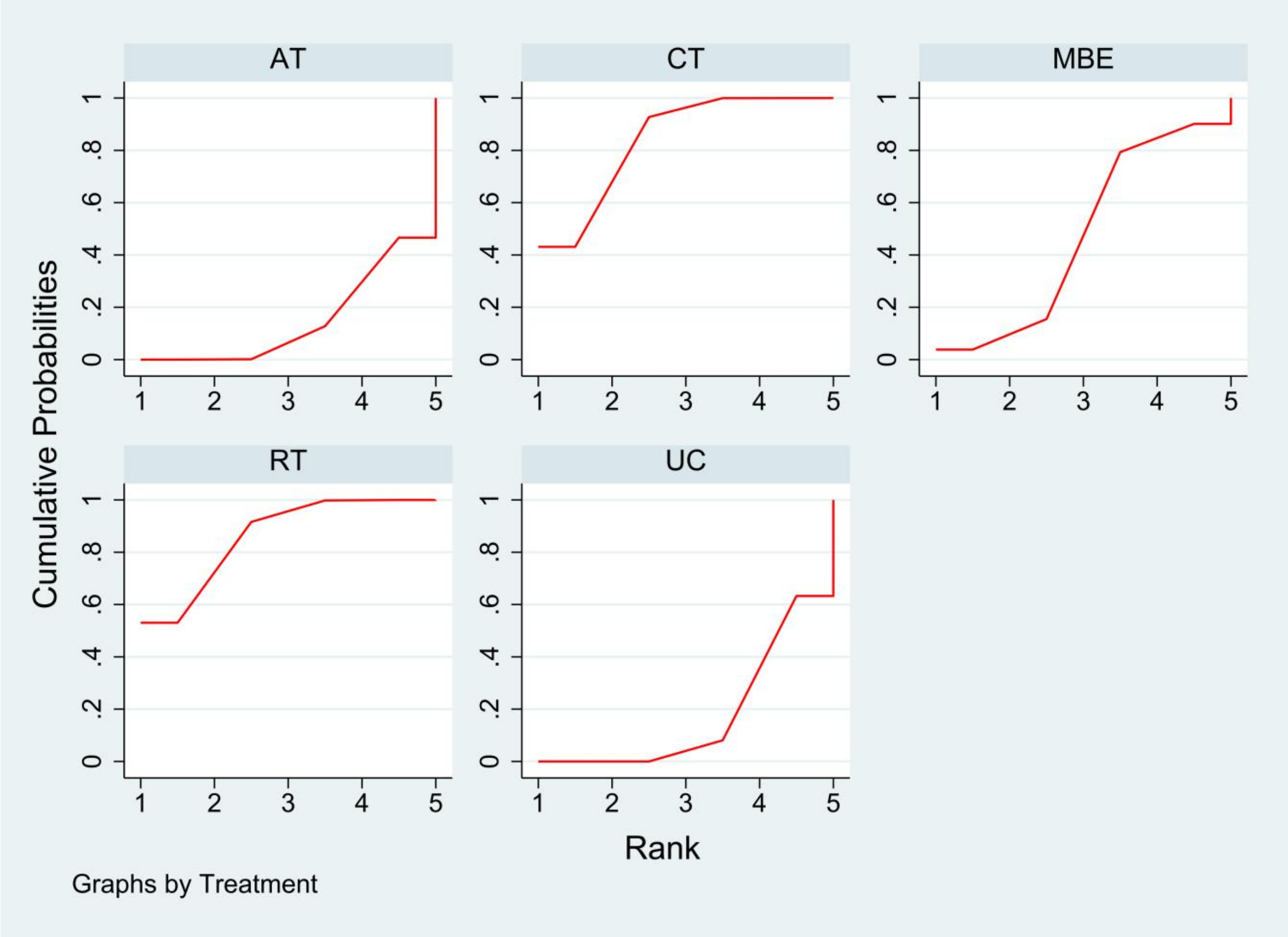


Abbreviations: FBG, fasting blood glucose;AT, aerobic training; RT, resistance training; CT, combined training (aerobic plus resistance training); MBE, mind-body exercise; UC, usual care.

**Table S6.4:** SUCRA values of different interventions for FBG.

| Treatment | SUCRA | PrBest | MeanRank |
|---|---|---|---|
| AT | 17. 9 | 0 | 4. 3 |
| CT | 84 | 43. 1 | 1. 6 |
| MBE | 47. 2 | 3. 8 | 3. 1 |
| RT | 86. 1 | 53 | 1. 6 |
| UC | 16. 8 | 0 | 4. 4 |

Abbreviations: SUCRA, surface under the cumulative ranking curve.

**Appendix7: League tables of relative treatment effects across exercise modalities**

**Table S7.1: eGFR.**Treatments are arranged along the diagonal. Comparisons between interventions should be read from column to row (i.e., column-defining intervention minus row-defining intervention). Estimates are presented as mean differences (MDs) with 95% confidence intervals (CIs). A positive MD indicates that the column intervention yields a higher eGFR than the row intervention, whereas a negative MD favors the row intervention. Significant results (95% CI excluding 0) are shown in bold.

| | | | |
|---|---|---|---|
| AT | | | |
| −1. 15（−6. 45, 4. 16） | CT | | |
| −8. 61（−15. 00, −2. 22） | −7. 46（−15. 15, 0. 24） | RT | |
| 2. 75（−1. 49, 7. 00） | 3. 90（−0. 45, 8. 25） | 11. 36（4. 47, 18. 25） | UC |

**Abbreviations:** AT, aerobic training; CT, combined training; RT, resistance training; UC, usual care; eGFR, estimated glomerular filtration rate; MD, mean difference; CI, confidence interval.

**Table S7.2: UAE(UACR or UAER).**Treatments are arranged along the diagonal. Comparisons between interventions should be read from column to row（column−defining intervention minus row−defining intervention）. Estimates are presented as standardized mean differences（SMDs）with 95% confidence intervals（CIs）. Because a lower value of UAE indicates clinical improvement, a negative SMD（< 0）favors the column intervention, whereas a positive SMD（> 0）favors the row intervention. Statistically significant results（95% CI excluding 0）are shown in bold.

| | | | | |
|---|---|---|---|---|
| AT | | | | |
| −0. 14 （−1. 21, 0. 92） | CT | | | |
| 0. 65 （−0. 85, 2. 15） | 0. 79 （−0. 75, 2. 33） | MBE | | |
| 0. 70 （−0. 54, 1. 95） | 0. 84 （−0. 80, 2. 48） | 0. 05 （−1. 89, 2. 00） | RT | |
| −0. 77 （−1. 59, 0. 06） | −0. 62 （−1. 52, 0. 28） | −1. 41 （−2. 67, −0. 16） | −1. 47 （−2. 96, 0. 03） | UC |

**Abbreviations**: AT, aerobic training; CT, combined training; MBE, mind−body exercise; RT, resistance training; UC, usual care; UAE, urinary albumin excretion; UACR, urinary albumin−to−creatinine ratio; UAER, urinary albumin excretion rate; CI, confidence interval.

**Table S7.3: HbA1c.** Treatments are arranged along the diagonal. Comparisons between interventions should be read from column to row（column−defining intervention minus row−defining intervention）. Estimates are presented as mean differences（MDs）with 95% confidence intervals（CIs）. Because a lower HbA1c level indicates better glycemic control, a negative MD（< 0）favors the column intervention, whereas a positive MD（> 0）favors the row intervention. Statistically significant results（95% CI excluding 0）are shown in bold.

| | | | | |
|---|---|---|---|---|
| AT | | | | |
| −0. 37（−1. 18, 0. 44） | CT | | | |
| −0. 70（−1. 96, 0. 57） | −0. 33（−1. 58, 0. 93） | MBE | | |
| 0. 58（−0. 40, 1. 55） | 0. 94（−0. 22, 2. 11） | 1. 27（−0. 22, 2. 77） | RT | |
| −1. 07（−1. 71, −0. 43） | −0. 70（−1. 33, −0. 08） | −0. 38（−1. 46, 0. 71） | −1. 65（−2. 67, −0. 62） | UC |

**Abbreviations**: AT, aerobic training; CT, combined training; MBE, mind−body exercise; RT, resistance training; UC, usual care; HbA1c, glycated hemoglobin; MD, mean difference; CI, confidence interval.

**Table S7.4: FBG.** Treatments are arranged along the diagonal. Comparisons between interventions should be read from column to row（column-defining intervention minus row-defining intervention）. Estimates are presented as mean differences（MDs）with 95% confidence intervals（CIs）. Because a lower FBG level indicates superior glycemic control, a negative MD（< 0）favors the column intervention, whereas a positive MD（> 0）favors the row intervention. Statistically significant results（95% CI excluding 0）are shown in bold.

| | | | | |
|---|---|---|---|---|
| AT | | | | |
| 1. 80（0. 76, 2. 85） | CT | | | |
| 0. 78（−0. 78, 2. 35） | −1. 02（−2. 53, 0. 49） | MBE | | |
| 1. 87（0. 58, 3. 16） | 0. 07（−1. 29, 1. 42） | 1. 09（−0. 59, 2. 77） | RT | |
| 0. 09（−0. 77, 0. 95） | −1. 71（−2. 51, −0. 91） | −0. 69（−1. 97, 0. 59） | −1. 78（−2. 92, −0. 64） | UC |

**Abbreviations**: AT, aerobic training; CT, combined training; MBE, mind-body exercise; RT, resistance training; UC, usual care; FBG, fasting blood glucose; MD, mean difference; CI, confidence interval.

**Appendix 8: CINeMA Assessment**

We used the CINeMA framework to assess the certainty of evidence for each network estimate based on the following criteria:

**A. Within-study bias:** The overall risk of bias for each study was evaluated using the Cochrane RoB 2 tool. We classified the overall risk of bias as 'low risk' when all assessed domains were rated as low risk of bias. A 'moderate risk' was assigned when at least one domain raised some concerns, but no domain was rated as high risk. A 'high risk' of bias was assigned if one or more domains were rated as high risk.

**Figure S8.1:** Risk of bias contribution by intervention group in eGFR

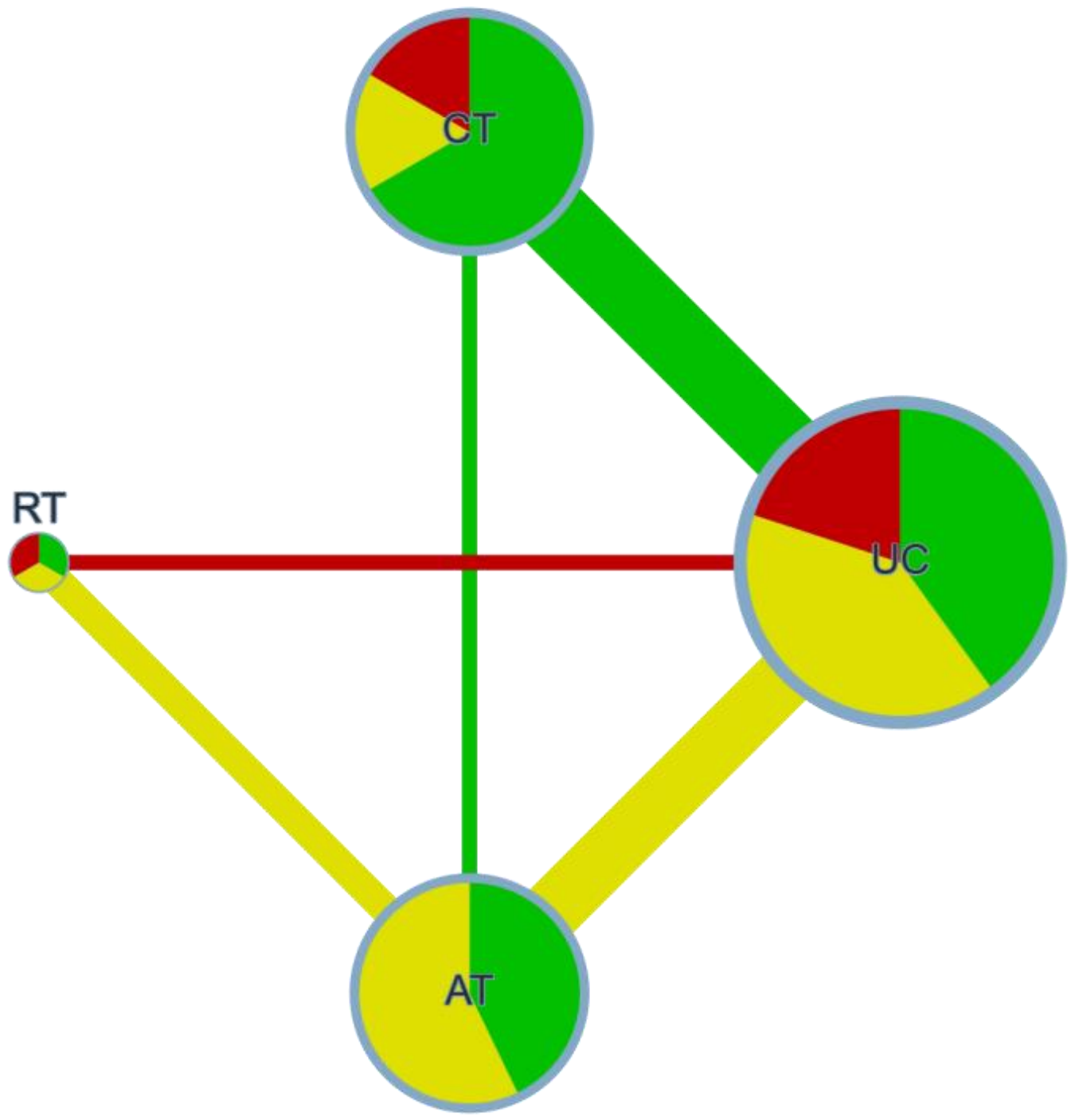


**Figure S8.2:** Overall risk of bias for eGFR was assessed by comparing intervention groups

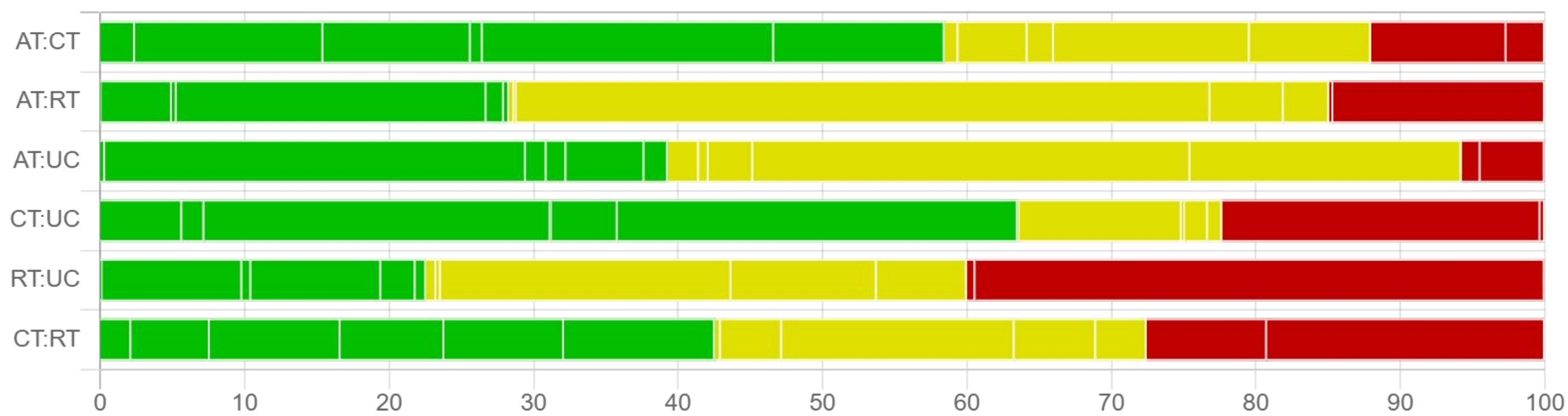

**Figure S8.3:** Risk of bias contribution by intervention group in UAE(UACR or UAER)

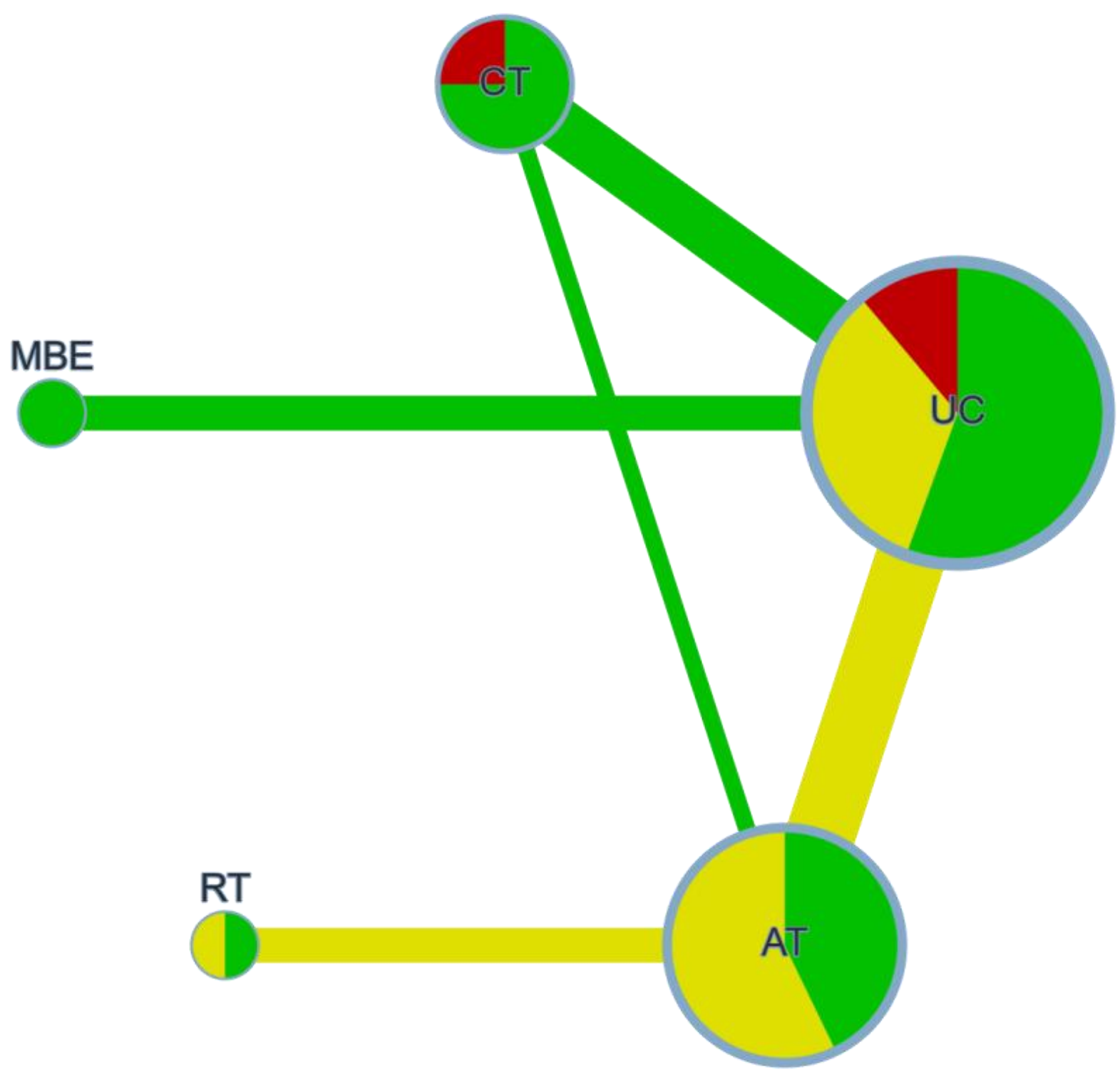


**Figure S8.4:** Overall risk of bias for UAE(UACR or UAER) was assessed by comparing intervention groups

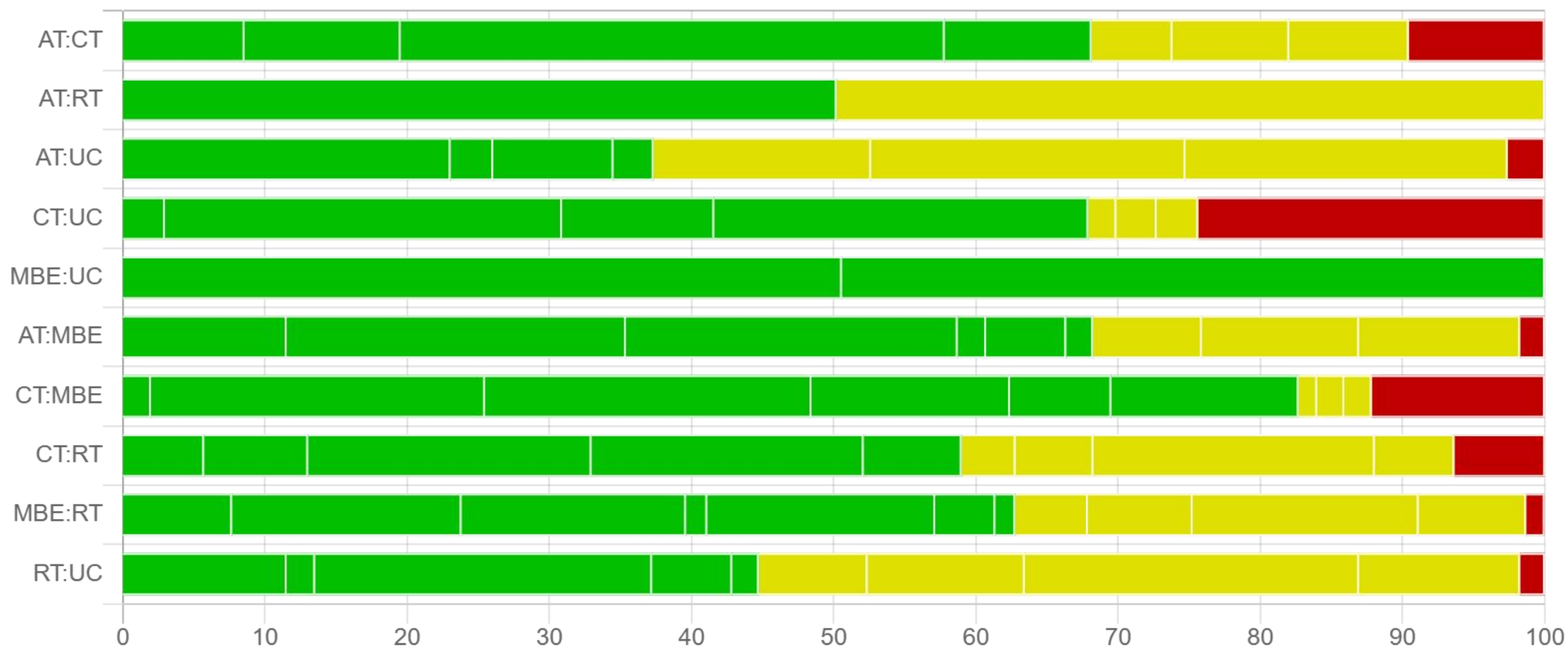

**Figure S8.5:** Risk of bias contribution by intervention group in HbA1c

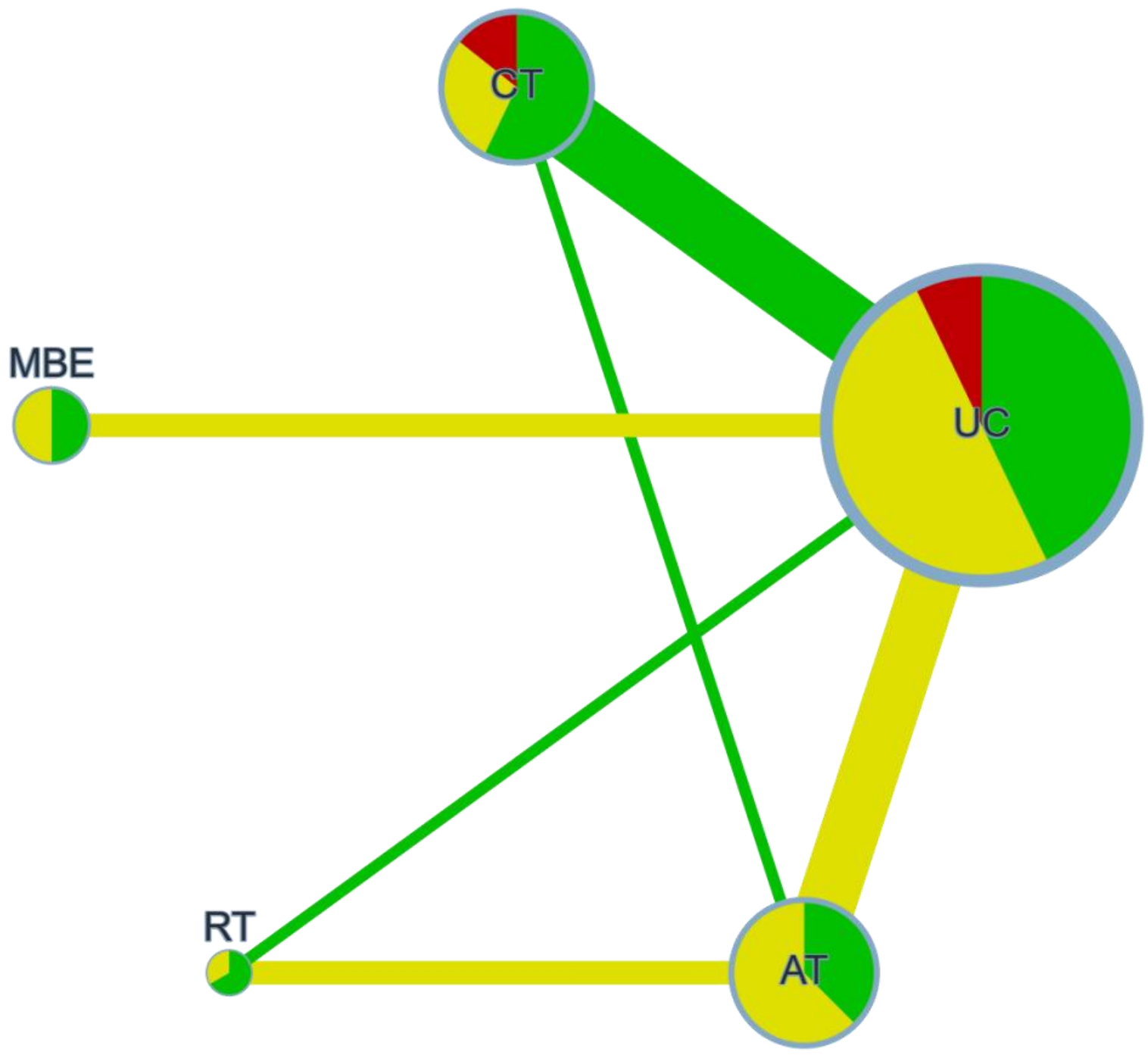


**Figure S8.6:** Overall risk of bias for HbA1c was assessed by comparing intervention groups

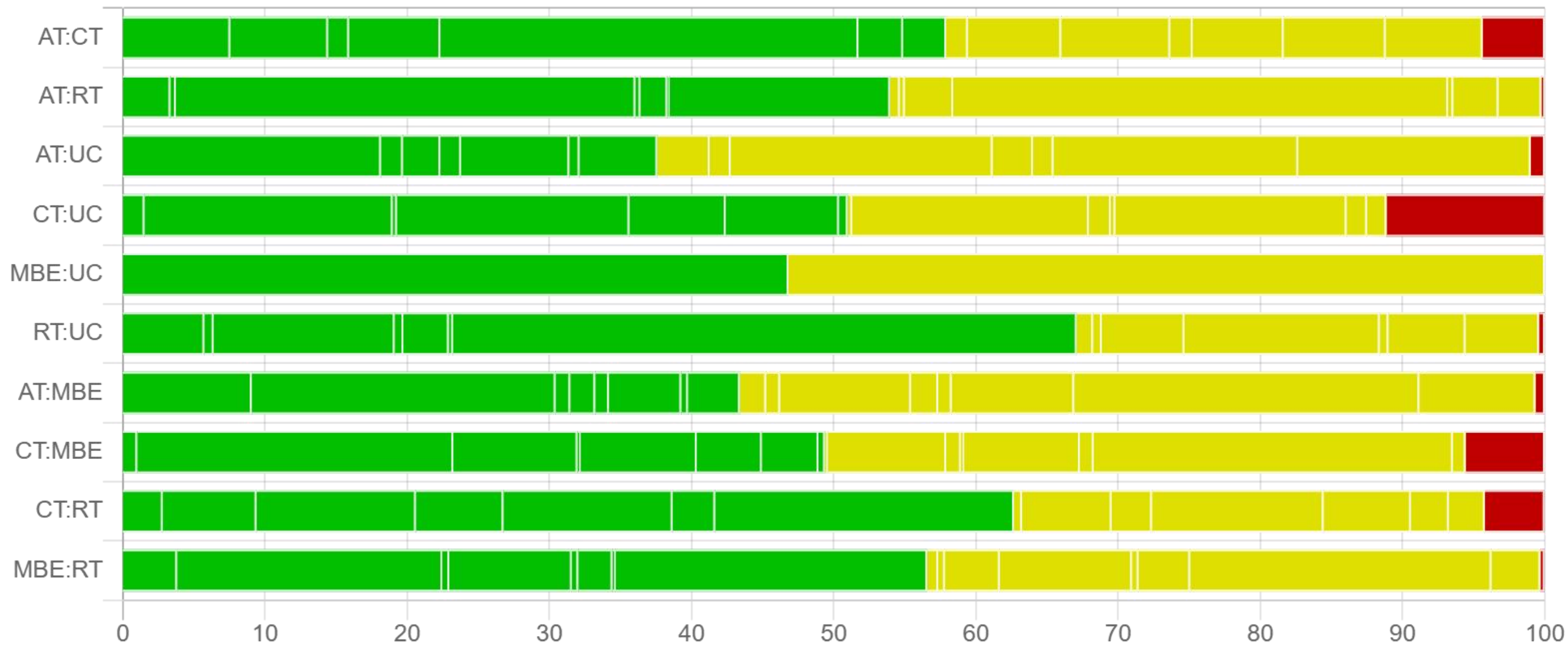

**Figure S8.7:** Risk of bias contribution by intervention group in FBG

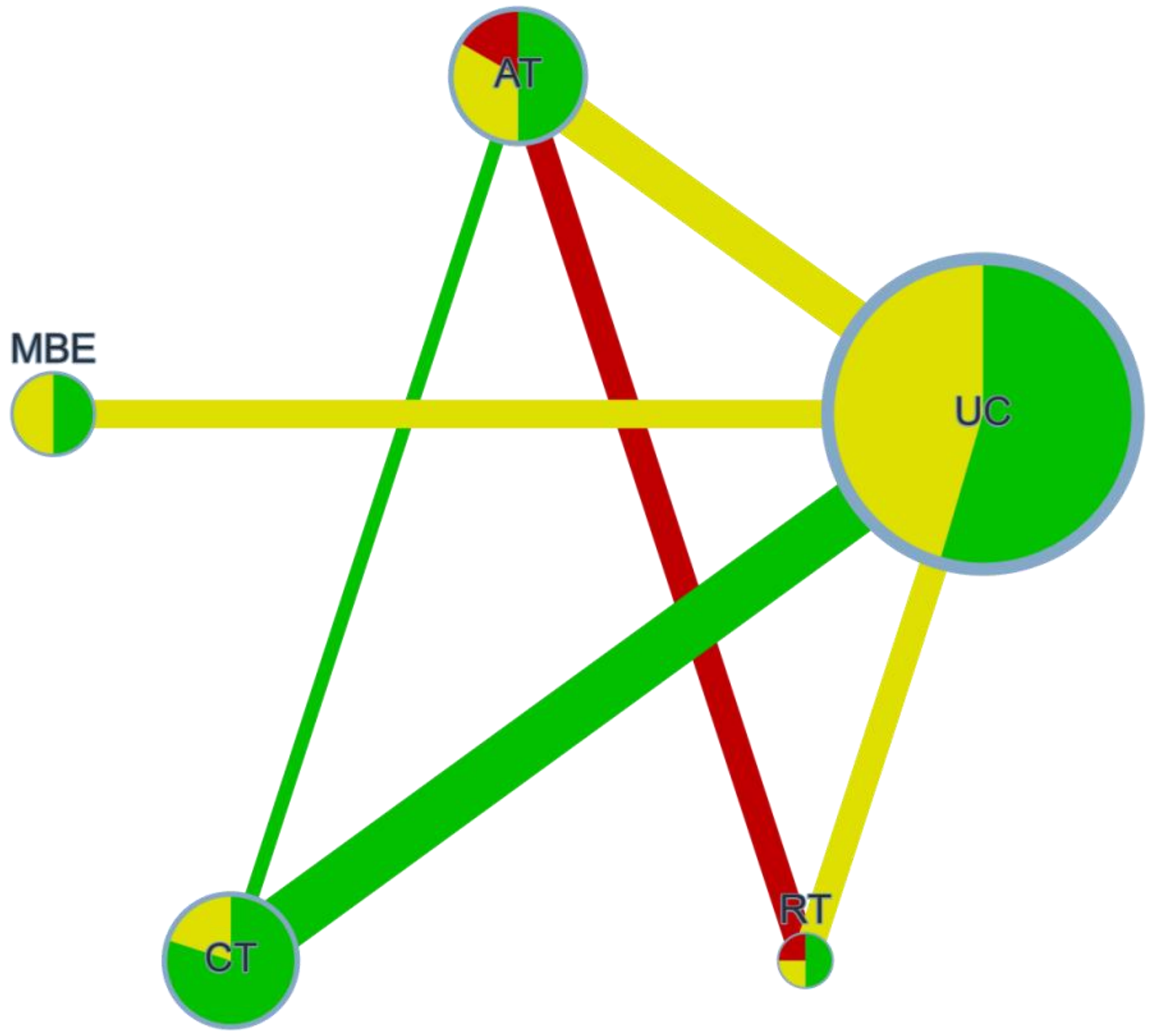


**Figure S8.8:** Overall risk of bias for FBG was assessed by comparing intervention groups

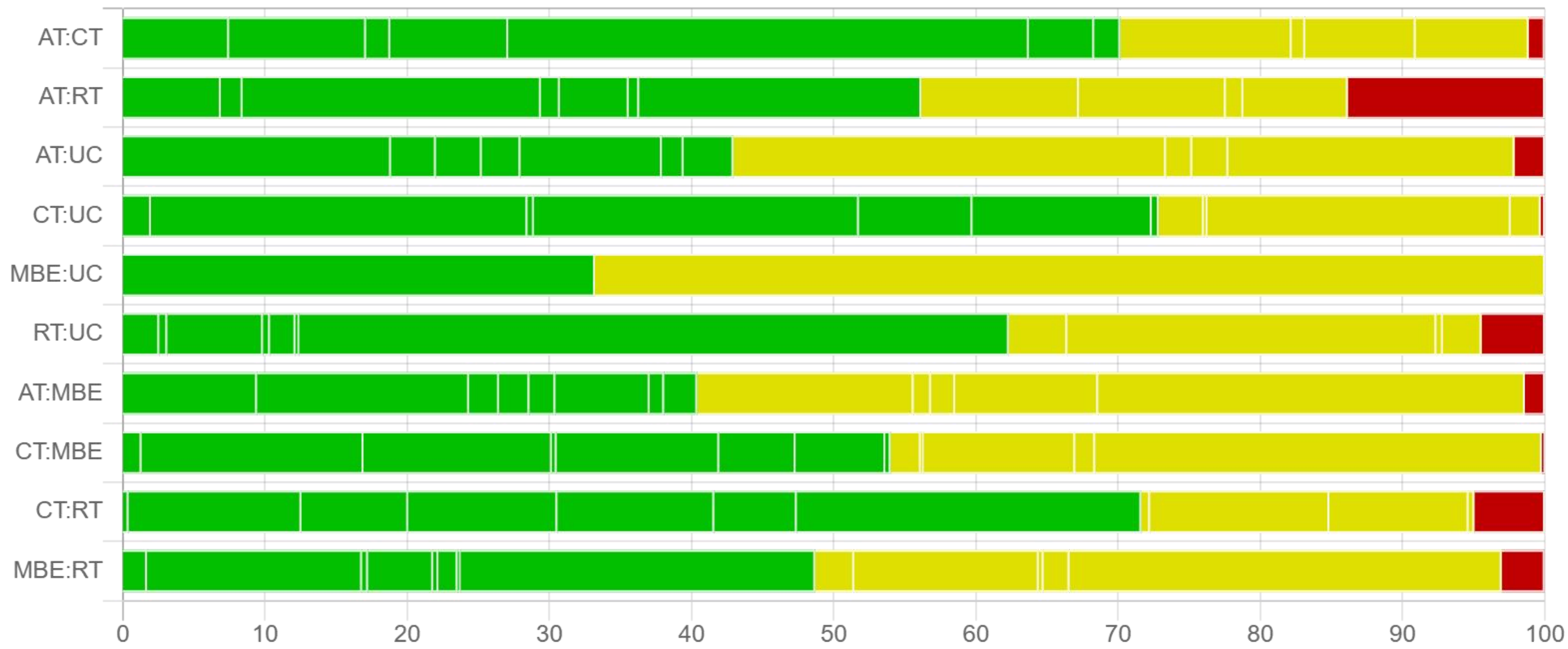

**Table S8.1:** Transitivity (Indirectness) Assessmen

| | Baseline variable (Mean ± SD) | | | | |
|---|---|---|---|---|---|
| Intervention | No. of Studies | Age(year) | Male % | HbA1c（%） | Weeks |
| AT vs UC | 5 | 59.50 ± 11.78* | 63.8% | 8.24 ± 1.06 | 24 (4–48) |
| RT vs UC | 2 | 58.38 ± 5.89 | 69.8% | 8.17 ± 1.29† | 10 (8–12) |
| CT vs UC | 7 | 62.26 ± 9.74 | 64.9%‡ | 8.59 ± 1.93§ | 24 (8–104) |
| MBE vs UC | 3 | 60.49 ± 8.51 | 54.6% | 6.75 ± 1.49¶ | 12 (8–24) |
| RT vs AT | 2 | 42.75 ± 12.97 | 49.7% | 8.83 ± 1.86 | 18 (12–24) |
| CT vs AT | 1 | 53.93 ± 7.78 | 66.0% | 8.89 ± 0.72 | 8 |

Note: Data are presented as pooled mean ± pooled standard deviation (calculated using Cochrane sample-size weighting formula) or percentage, unless otherwise indicated. Weeks are presented as mean ± SD (or median and range).

* Reported in 4 studies (n=310).

† Reported in 1 study (n=86).

‡ Reported in 6 studies (n=558).

§ Reported in 6 studies (n=558).

¶ Reported in 2 studies (n=326).

Transitivity statement: Key clinical effect modifiers including baseline age, sex ratio, glycemic control (HbA1c), and intervention duration were reasonably balanced across the pairwise comparisons, confirming that the transitivity assumption was generally met for the network meta-analysis.

**Abbreviations:** AT, aerobic training; CT, combined training; RT, resistance training; UC, usual care; eGFR, estimated glomerular filtration rate; MBE, mind-body exercise.

**Imprecision:** Imprecision was evaluated using the CINeMA web application by examining whether the 95% confidence intervals (CIs) of the relative effect estimates crossed the pre-specified margins of clinical equivalence.

**Heterogeneity:** Heterogeneity was assessed by comparing the 95% CIs with the 95% prediction intervals (PIs) relative to the predefined thresholds of clinical significance. We applied the same clinical reasoning framework used for imprecision and adopted the automated judgments provided by the CINeMA platform (https://cinema.ispm.unibe.ch/) to determine the level of concern regarding heterogeneity.

**Inconsistency:** Incoherence between direct and indirect evidence was assessed using the node-splitting method (Appendix 5). No significant inconsistency was detected for the majority of comparisons ($P > 0.05$), indicating an acceptable level of overall network coherence.

**Table S8.2:** CINeMA assessment of confidence in the evidence for eGFR

| Comparison | Within-study bias | Reporting bias | Indirectness | Imprecision | Heterogeneity | Incoherence | Confidence rating |
|---|---|---|---|---|---|---|---|
| AT:CT | Some concerns | Low risk | No concerns | Major concerns | No concerns | No concerns | Low |
| AT:RT | Some concerns | Low risk | No concerns | No concerns | Major concerns | No concerns | Low |
| AT:UC | Some concerns | Low risk | No concerns | Major concerns | No concerns | No concerns | Low |
| CT:UC | Some concerns | Low risk | No concerns | No concerns | Major concerns | No concerns | Low |
| RT:UC | Major concerns | Low risk | No concerns | Some concerns | No concerns | No concerns | Low |
| CT:RT | Some concerns | Low risk | No concerns | No concerns | Major concerns | Major concerns | Low |

**Abbreviations:** AT, aerobic training; CT, combined training; RT, resistance training; UC, usual care; eGFR, estimated glomerular filtration rate.

**Table S8.3:** CINeMA assessment of confidence in the evidence for UAE(UACR or UAER)

| Comparison | Within-study bias | Reporting bias | Indirectness | Imprecision | Heterogeneity | Incoherence | Confidence rating |
|---|---|---|---|---|---|---|---|
| AT : CT | No concerns | Low risk | No concerns | Major concerns | No concerns | No concerns | Low |
| AT : RT | No concerns | Low risk | No concerns | Major concerns | No concerns | Major concerns | Low |
| AT : UC | Some concerns | Low risk | No concerns | Major concerns | No concerns | No concerns | Low |
| CT : UC | Some concerns | Low risk | No concerns | Major concerns | No concerns | No concerns | Low |
| MBE : UC | No concerns | Low risk | No concerns | No concerns | Major concerns | Major concerns | Low |
| AT : MBE | No concerns | Low risk | No concerns | Major concerns | No concerns | Major concerns | Low |
| CT : MBE | No concerns | Low risk | No concerns | No concerns | Major concerns | Major concerns | Low |
| CT : RT | No concerns | Low risk | No concerns | Major concerns | No concerns | Major concerns | Low |
| MBE : RT | No concerns | Low risk | No concerns | Major concerns | No concerns | Major concerns | Low |
| RT : UC | Some concerns | Low risk | No concerns | No concerns | Major concerns | Major concerns | Low |

**Abbreviations:** AT, aerobic training; CT, combined training; MBE, mind-body exercise; RT, resistance training; UC, usual care; UAE, urinary albumin excretion; UACR, urinary albumin-to-creatinine ratio; UAER, urinary albumin excretion rate.

**Table S8.4:** CINeMA assessment of confidence in the evidence for HbA1c

| Comparison | Within−study bias | Reporting bias | Indirectness | Imprecision | Heterogeneity | Incoherence | Confidence rating |
|---|---|---|---|---|---|---|---|
| AT : CT | No concerns | Low risk | No concerns | Major concerns | No concerns | No concerns | Low |
| AT : RT | No concerns | Low risk | No concerns | Major concerns | No concerns | No concerns | Low |
| AT : UC | Some concerns | Low risk | No concerns | No concerns | Major concerns | No concerns | Low |
| CT : UC | Some concerns | Low risk | No concerns | Major concerns | No concerns | No concerns | Low |
| MBE : UC | Some concerns | Low risk | No concerns | Major concerns | No concerns | Major concerns | Low |
| RT : UC | No concerns | Low risk | No concerns | No concerns | Major concerns | No concerns | Low |
| AT : MBE | Some concerns | Low risk | No concerns | Major concerns | No concerns | Major concerns | Low |
| CT : MBE | Some concerns | Low risk | No concerns | Major concerns | No concerns | Major concerns | Low |
| CT : RT | No concerns | Low risk | No concerns | Major concerns | No concerns | Major concerns | Low |
| MBE : RT | No concerns | Low risk | No concerns | Major concerns | No concerns | Major concerns | Low |

**Abbreviations:** AT, aerobic training; CT, combined training; MBE, mind−body exercise; RT, resistance training; UC, usual care; HbA1c, glycated hemoglobin.

**Table S8.5:** CINeMA assessment of confidence in the evidence for FBG

| Comparison | Within-study bias | Reporting bias | Indirectness | Imprecision | Heterogeneity | Incoherence | Confidence rating |
|---|---|---|---|---|---|---|---|
| AT : CT | Some concerns | Low risk | No concerns | No concerns | No concerns | No concerns | moderate |
| AT : RT | Some concerns | Low risk | No concerns | No concerns | Major concerns | Major concerns | Low |
| AT : UC | Some concerns | Low risk | No concerns | Major concerns | No concerns | No concerns | Low |
| CT : UC | Some concerns | Low risk | No concerns | No concerns | No concerns | No concerns | moderate |
| MBE : UC | Some concerns | Low risk | No concerns | Major concerns | No concerns | Major concerns | Low |
| RT : UC | No concerns | Low risk | No concerns | No concerns | No concerns | Major concerns | Low |
| AT : MBE | Some concerns | Low risk | No concerns | Major concerns | No concerns | Major concerns | Low |
| CT : MBE | No concerns | Low risk | No concerns | No concerns | Major concerns | Major concerns | Low |
| CT : RT | No concerns | Low risk | No concerns | Major concerns | No concerns | Major concerns | Low |
| MBE : RT | Some concerns | Low risk | No concerns | Major concerns | No concerns | Major concerns | Low |

**Abbreviations:** AT, aerobic training; CT, combined training; MBE, mind-body exercise; RT, resistance training; UC, usual care; FBG, fasting blood glucose.

**Appendix 9: Funnel plots**

**Figure S9.1:** Funnel plot of eGFR

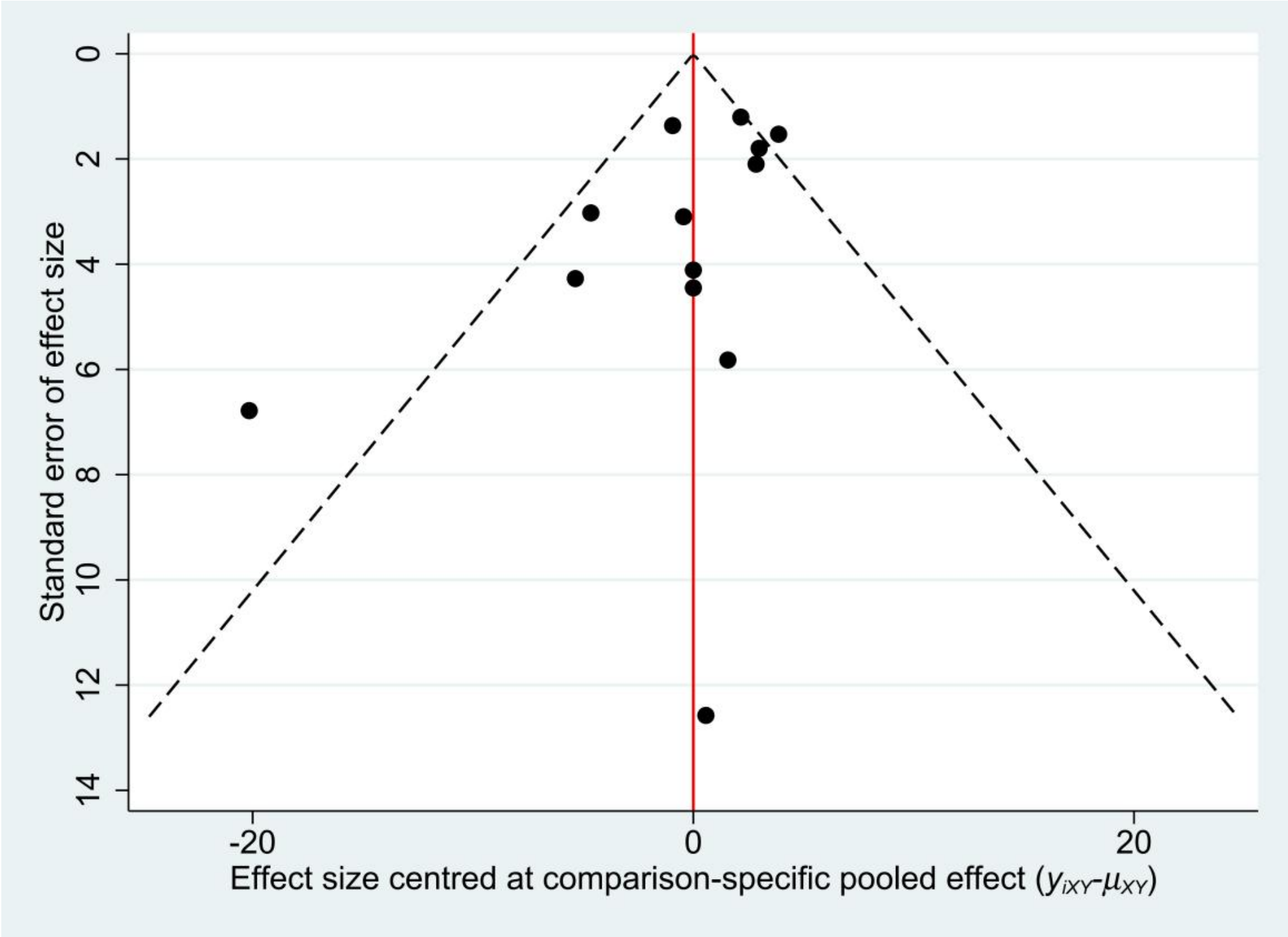


**Figure S9.2:** Funnel plot of UAE(UACR or UAER)

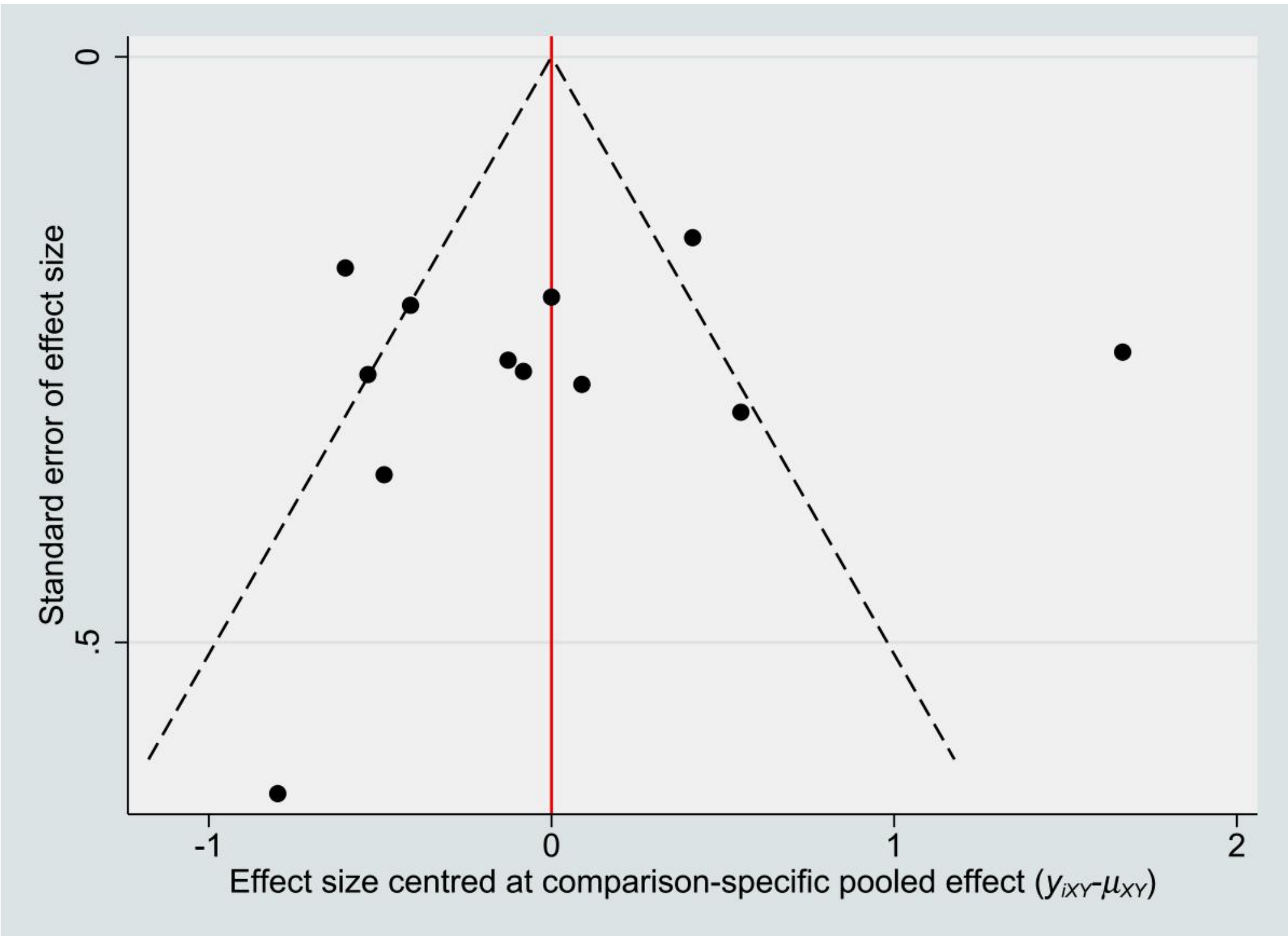

**Figure S9.3:** Funnel plot of HbA1c

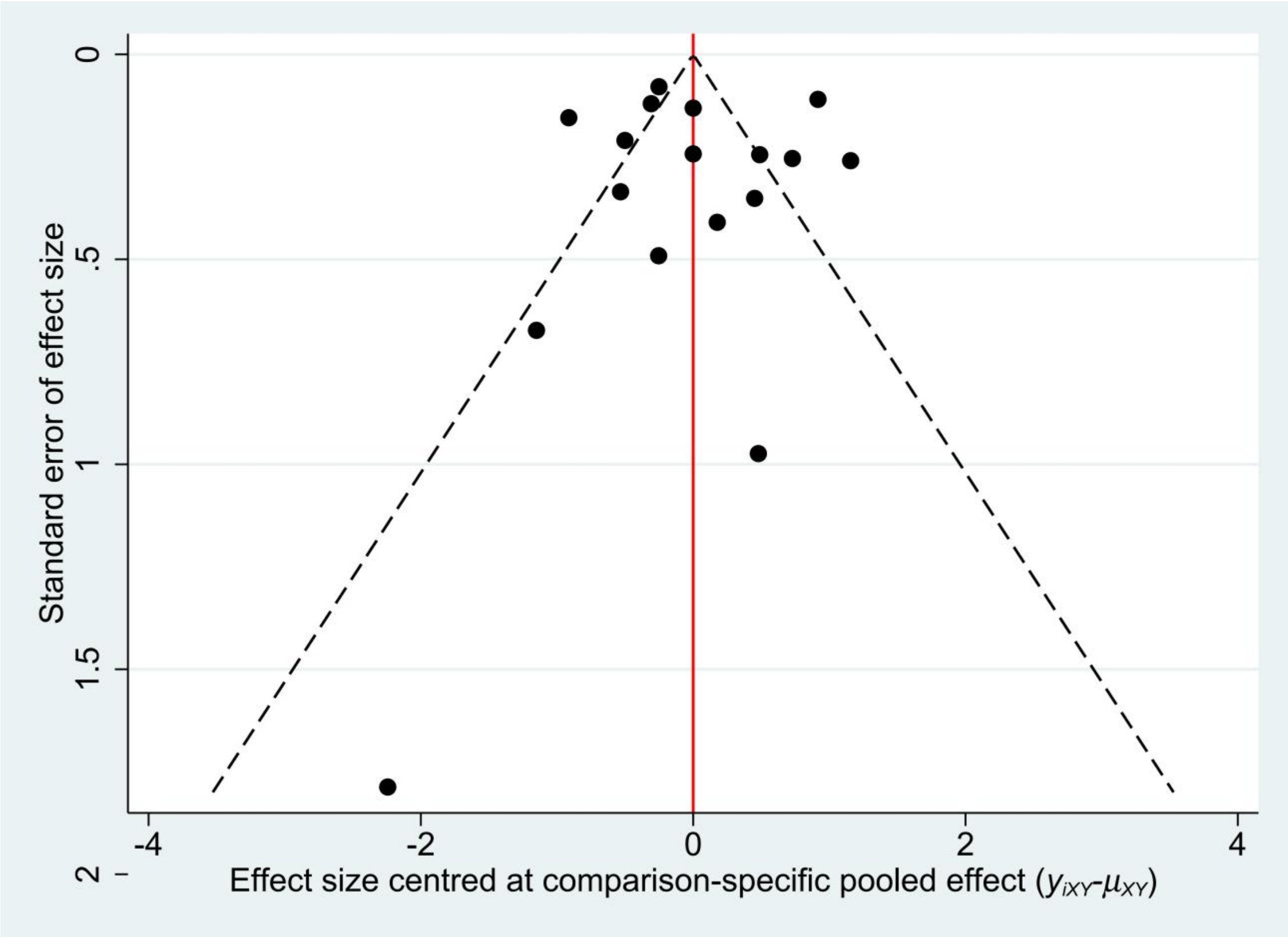

**Figure S9.4:** Funnel plot of FBG

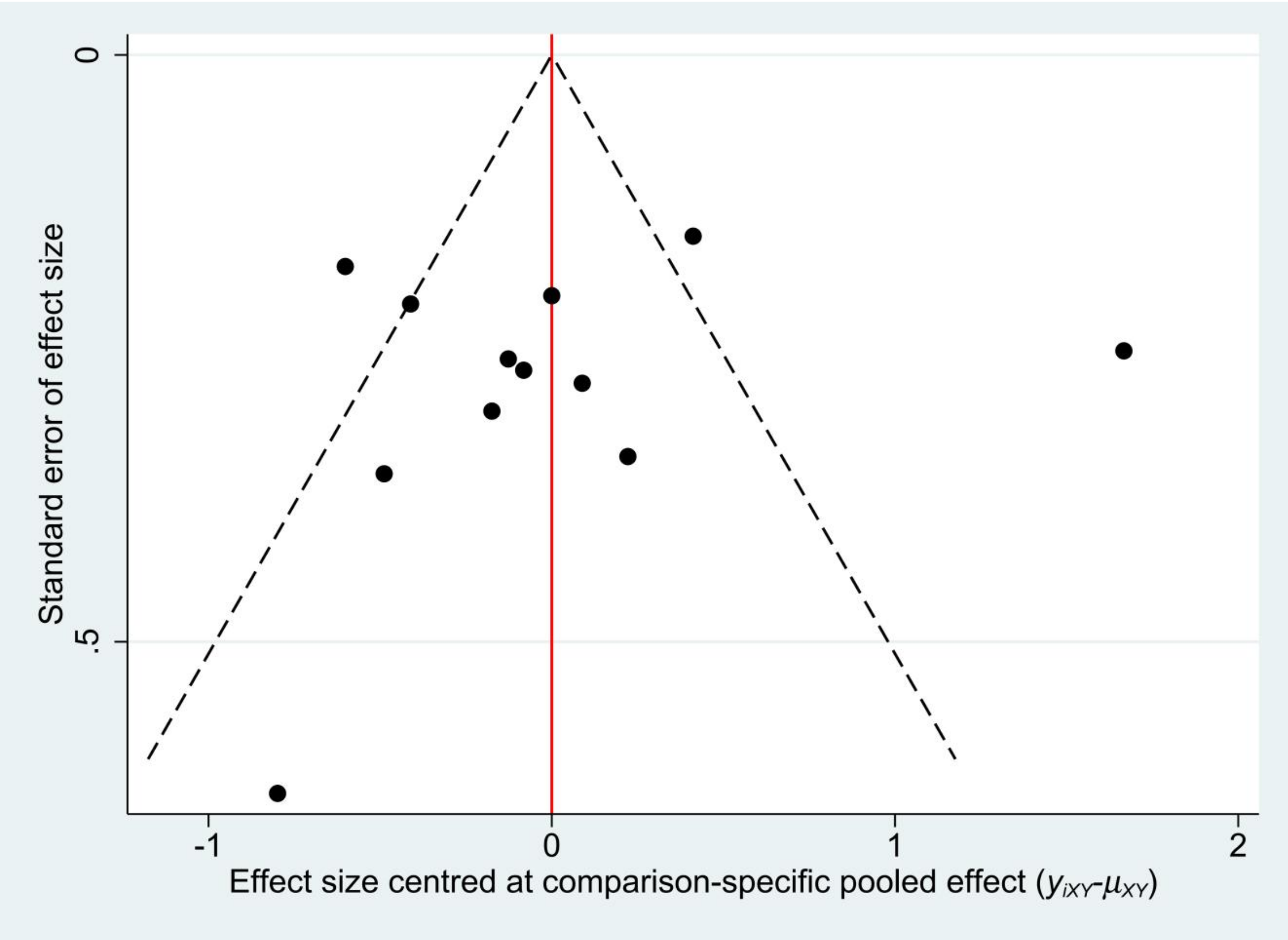

**Appendix 10: Sensitivity analyses**

**Figure S10.1:** Flow diagram of sensitivity analyses.

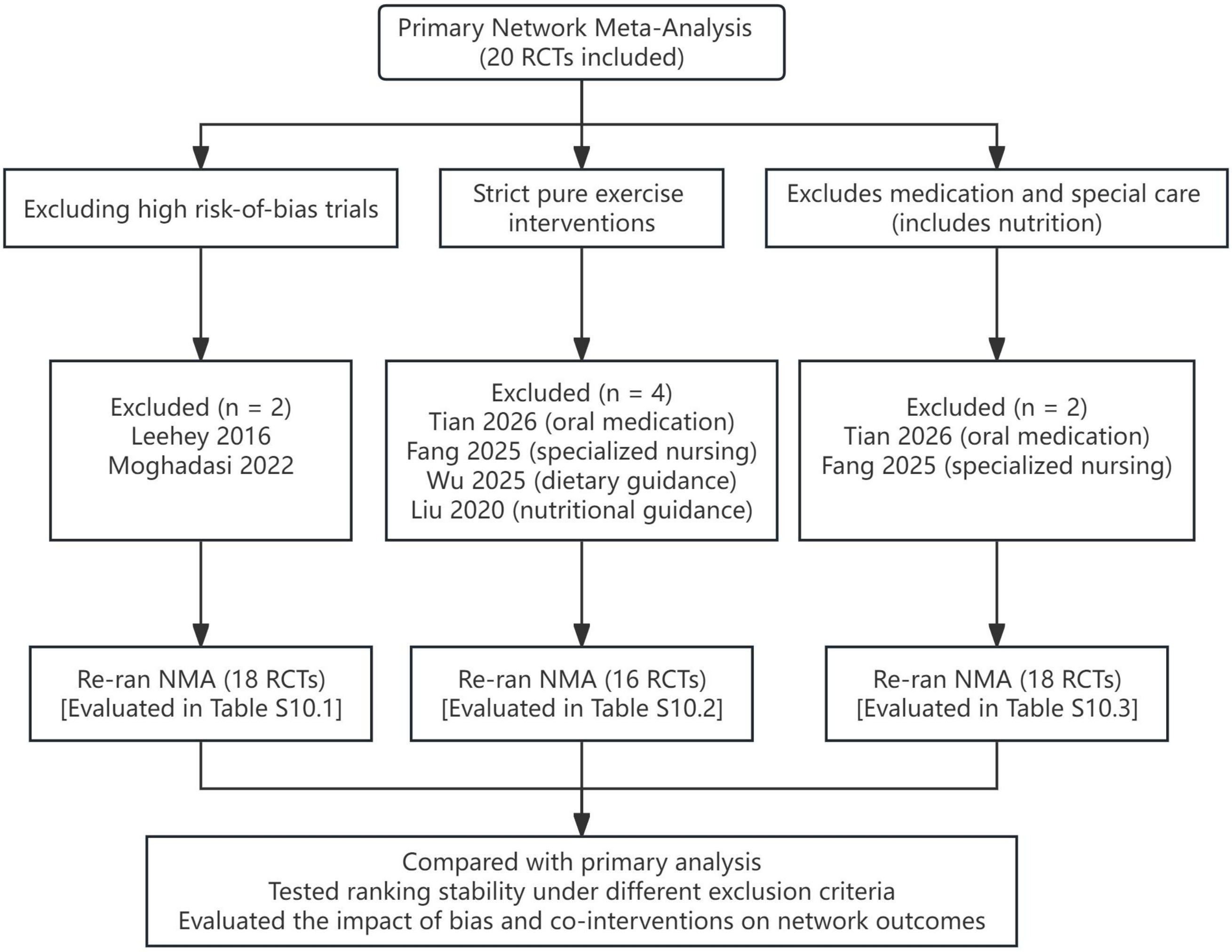

**Table S10.1:** Sensitivity analysis: Excluding high risk-of-bias trials

| Exercise modality | eGFR | | UAE(UACR or UAER) | | HbA1c | | FBG | |
|---|---|---|---|---|---|---|---|---|
| | Primary | Sensitivity | Primary | Sensitivity | Primary | Sensitivity | Primary | Sensitivity |
| **AT vs UC** | 2.75 (-1.49,7.00) | 2.16 (-2.59,6.92) | -0.77 (-1.59,0.06) | -0.82 (-1.65,0.01) | -1.07 (-1.71,-0.43) | -1.10 (-1.73,-0.48) | 0.09 (-0.77,0.95) | -0.19 (-0.84,0.47) |
| **CT vs UC** | 3.90 (-0.45,8.25) | 4.81 (-0.53,10.15) | -0.62 (-1.52,0.28) | -0.87 (-1.91,0.17) | -0.70 (-1.33,-0.08) | -0.85 (-1.49,-0.21) | -1.71 (-2.51,-0.91) | -1.75 (-2.35,-1.16) |
| **RT vs UC** | 11.36 (4.47,18.25) | 8.24 (-1.17,17.64) | -1.47 (-2.96,0.03) | -1.52 (-3.02,-0.02) | -1.65 (-2.67,-0.62) | -1.67 (-2.67,-0.67) | -1.78 (-2.92,-0.64) | -1.24 (-2.16,-0.32) |
| **MBE vs UC** | NA | NA | -1.41 (-2.67,-0.16) | -1.41 (-2.67,-0.16) | -0.38 (-1.46,0.71) | -0.37 (-1.43,0.68) | -0.69 (-1.97,0.59) | -0.58 (-1.56,0.40) |

**Abbreviations:** AT, aerobic training; CT, combined training; RT, resistance training; MBE, mind-body exercise; UC, usual care; eGFR, estimated glomerular filtration rate; UAE, urinary albumin excretion; UACR, urinary albumin-to-creatinine ratio; UAER, urinary albumin excretion rate; HbA1c, glycated hemoglobin; FBG, fasting blood glucose; MD, mean difference; CI, confidence interval.

**Note:** Data are presented as mean difference (MD) with 95% confidence intervals against usual care (UC). The sensitivity analysis was performed by excluding trials rated as having a high overall risk of bias based on the Cochrane RoB 2 tool.

**Table S10.2**: Sensitivity analysis: Excluding confounding co-interventions to evaluate pure exercise effects

| Exercise modality | eGFR | | UAE(UACR or UAER) | | HbA1c | | FBG | |
|---|---|---|---|---|---|---|---|---|
| | Primary | Sensitivity | Primary | Sensitivity | Primary | Sensitivity | Primary | Sensitivity |
| AT vs UC | 2.75 (-1.49,7.00) | 1.92 (-2.87,6.71) | -0.77 (-1.59,0.06) | -0.77 (-1.59,0.06) | -1.07 (-1.71,-0.43) | -1.14 (-2.01,-0.27) | 0.09 (-0.77,0.95) | -0.09 (-0.90,0.71) |
| CT vs UC | 3.90 (-0.45,8.25) | 3.81 (-0.72,8.33) | -0.62 (-1.52,0.28) | -0.62 (-1.52,0.28) | -0.70 (-1.33,-0.08) | -0.60 (-1.39,0.19) | -1.71 (-2.51,-0.91) | -1.97 (-2.76,-1.19) |
| RT vs UC | 11.36 (4.47,18.25) | 10.81 (3.74,17.88) | -1.47 (-2.96,0.03) | -1.47 (-2.96,0.03) | -1.65 (-2.67,-0.62) | -1.82 (-3.43,-0.21) | -1.78 (-2.92,-0.64) | -2.22 (-3.61,-0.84) |
| MBE vs UC | NA | NA | -1.41 (-2.67,-0.16) | -1.41 (-2.67,-0.16) | -0.38 (-1.46,0.71) | -0.79 (-2.62,1.04) | -0.69 (-1.97,0.59) | -1.71 (-3.51,0.09) |

**Abbreviations:** AT, aerobic training; CT, combined training; RT, resistance training; MBE, mind-body exercise; UC, usual care; MD, mean difference; CI, confidence interval.

**Note:** Data are MDs (95% CI) versus UC. Sensitivity analysis excludes four trials with potential confounding co-interventions: Tian et al. (2026) [oral medication: Qizhi Yishen capsule], Fang et al. (2025) [specialized nursing: ITHBC model], Wu et al. (2025) [dietary guidance], and Liu et al. (2020) [nutritional guidance].

**Note:** The effect estimates for UAE in sensitivity analyses are identical to the primary analysis because none of the excluded trials contributed data to this specific endpoint.

**Table S10.3**: Sensitivity analysis: Excluding medication and nursing while retaining dietary guidance

| Exercise modality | eGFR | | UAE(UACR or UAER) | | HbA1c | | FBG | |
|---|---|---|---|---|---|---|---|---|
| | Primary | Sensitivity | Primary | Sensitivity | Primary | Sensitivity | Primary | Sensitivity |
| **AT vs UC** | 2.75 (-1.49,7.00) | 2.75 (-1.49,7.00) | -0.77 (-1.59,0.06) | -0.77 (-1.59,0.06) | -1.07 (-1.71,-0.43) | -1.10 (-1.82,-0.37) | 0.09 (-0.77,0.95) | -0.01 (-0.86,0.84) |
| **CT vs UC** | 3.90 (-0.45,8.25) | 3.90 (-0.45,8.25) | -0.62 (-1.52,0.28) | -0.62 (-1.52,0.28) | -0.70 (-1.33,-0.08) | -0.70 (-1.37,-0.03) | -1.71 (-2.51,-0.91) | -1.73 (-2.50,-0.96) |
| **RT vs UC** | 11.36 (4.47,18.25) | 11.36 (4.47,18.25) | -1.47 (-2.96,0.03) | -1.47 (-2.96,0.03) | -1.65 (-2.67,-0.62) | -1.77 (-3.24,-0.31) | -1.78 (-2.92,-0.64) | -2.23 (-3.67,-0.79) |
| **MBE vs UC** | NA | NA | -1.41 (-2.67,-0.16) | -1.41 (-2.67,-0.16) | -0.38 (-1.46,0.71) | -0.79 (-2.50,0.92) | -0.69 (-1.97,0.59) | -1.71 (-3.65,0.23) |

**Abbreviations:** AT, aerobic training; CT, combined training; RT, resistance training; MBE, mind-body exercise; UC, usual care; MD, mean difference; CI, confidence interval.

**Note:** Data are MDs (95% CI) versus UC. Pragmatic scenario excludes trials with active pharmacological or specialized nursing models (Tian et al., 2026; Fang et al., 2025) while retaining guideline-directed nutritional interventions (Wu et al., 2025; Liu et al., 2020).

**Note:** The effect estimates for eGFR and UAE in sensitivity analyses are identical to the primary analysis because none of the excluded trials contributed data to this specific endpoint.

## Appendix 11: Subgroup analysis

### Table S11.1: Subgroup analysis by intervention duration

| Exercise modality (vs. UC) | eGFR | | UAE(UACR or UAER) | | HbA1c | | FBG | |
|---|---|---|---|---|---|---|---|---|
| | ≤12weeks | ＞12weeks | ≤12weeks | ＞12weeks | ≤12weeks | ＞12weeks | ≤12weeks | ＞12weeks |
| AT | 3.82 (0.09,7.55) | -1.49 (-14.19,11.21) | -0.38 (-1.28,0.53) | -1.16 (-2.46,0.15) | -1.21 (-2.03,-0.38) | -1.26 (-2.94,0.43) | 0.45 (-1.28,2.17) | -0.26 (-0.97,0.45) |
| CT | 1.55 (-2.77,5.88) | 7.02 (-1.32,15.36) | -0.96 (-1.85,-0.07) | -0.04 (-1.60,1.52) | -1.06 (-1.80,-0.31) | 0.00 (-1.65,1.65) | -1.50 (-2.77,-0.23) | -2.39 (-3.74,-1.04) |
| RT | 11.94 (5.95,17.93) | 5.91 (-16.15,27.97) | -1.41 (-2.24,-0.57) | -1.94 (-4.49,0.60) | -1.58 (-2.72,-0.43) | -0.03 (-2.85,2.78) | -1.73 (-3.38,-0.09) | -2.76 (-4.76,-0.76) |
| MBE | NA | NA | -0.99 (-2.47,0.48) | NA | -0.79 (-2.33,0.75) | -1.72 (-5.03,1.59) | -1.71 (-4.32,0.91) | 0.00 (-0.65,0.65) |

**Abbreviations:** AT, aerobic training; CT, combined training; RT, resistance training; MBE, mind-body exercise; UC, usual care; eGFR, estimated glomerular filtration rate; UAE, urinary albumin excretion; UACR, urinary albumin-to-creatinine ratio; UAER, urinary albumin excretion rate; HbA1c, glycated hemoglobin; FBG, fasting blood glucose; MD, mean difference; CI, confidence interval; NA, not available.

**Note:** Data are presented as mean differences (MDs) [or standardized mean differences for UAE] with 95% confidence intervals (CIs) versus usual care (UC). Subgroups were categorized by intervention duration into short-term (≤ 12 weeks) and medium-to-long-term (> 12 weeks) interventions. NA indicates that the specific exercise modality could not be estimated due to the absence of eligible trials in that duration subgroup.

**Appendix 12: Exploratory Analyses**

**Figure S12.1: Exploratory network meta-analysis of systolic blood pressure (SBP)**

The exploratory network for SBP synthesized 6 randomized controlled trials (439 participants) evaluating three active exercise modalities (aerobic training [AT], combined training [CT], and mind–body exercise [MBE]) against usual care (UC) in non-dialysis diabetic kidney disease. This endpoint was designated as an exploratory secondary analysis because the network geometry is strictly star-shaped around usual care and completely lacks closed triangular loops between active exercise regimens (Panel A), precluding formal assessment of local incoherence via the node-splitting method.

As illustrated in the forest plot (Panel B), mind–body exercise (MBE) produced the most substantial reduction in SBP compared with usual care (mean difference [MD] –15.70 mmHg, 95% CI –20.57 to –10.82; moderate certainty), followed by aerobic training (MD –5.50 mmHg, 95% CI –10.55 to –0.44; low certainty). In contrast, combined training showed no statistically significant difference relative to usual care (MD 2.74 mmHg, 95% CI –4.02 to 9.50; low certainty). While these exploratory findings highlight the potential hemodynamic advantages of mind–body exercise in mitigating cardiovascular risk, the conclusions warrant cautious interpretation owing to the sparse geometric structure and reliance on indirect active contrasts.

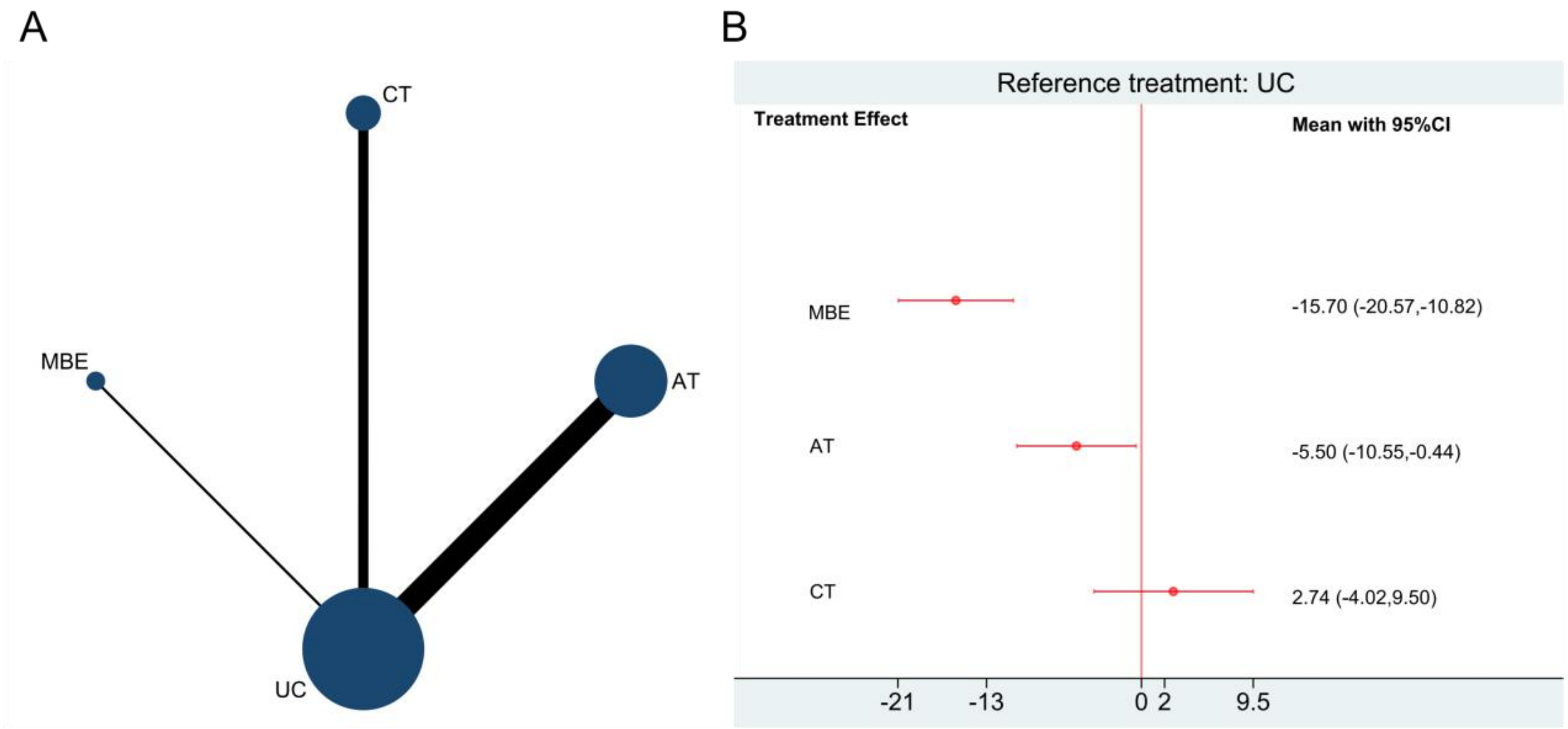


Note: In panel B, a mean difference (MD) < 0 indicates a favourable effect of the specific exercise modality in reducing SBP compared with usual care (UC). Node sizes in panel A reflect total sample sizes, and edge thicknesses correspond to the number of direct comparison trials.

Abbreviations: AT, aerobic training; CI, confidence interval; CT, combined training (aerobic plus resistance); MBE, mind–body exercise; MD, mean difference; SBP, systolic blood pressure; UC, usual care.

**Figure S12.2: Exploratory network meta-analysis of serum creatinine (SCr)**

The exploratory network for SCr synthesized 9 randomized controlled trials (590 participants) investigating aerobic training (AT), combined training (CT), and resistance training (RT) against usual care (UC) in non-dialysis diabetic kidney disease (Panel A). This metric was relegated to an exploratory secondary analysis due to combined methodological, statistical, and physiological considerations. First and foremost, eGFR is mathematically derived from circulating SCr via standardized filtration equations (such as CKD-EPI and MDRD) after adjusting for crucial demographic covariates (e.g., age, sex); thus, retaining raw SCr in the primary text would introduce substantial collinearity and outcome redundancy relative to eGFR. Second, structured exercise—particularly resistance training—stimulates muscle protein turnover and increases lean skeletal muscle mass, naturally elevating endogenous creatinine generation and making raw circulating SCr an inherently confounded surrogate of true renal function. Finally, from a network topology perspective, the SCr geometry is completely acyclic and lacks closed loops (RT was compared exclusively against AT, while CT was compared solely against UC), precluding statistical incoherence evaluation via the node-splitting method and necessitating reliance on indirect contrasts.

As presented in the forest plot (Panel B), combined training (CT) was the only modality to produce a statistically significant reduction in SCr compared with usual care (mean difference [MD] –15.08 μmol/L, 95% CI –24.81 to –5.36; low certainty). Neither aerobic training (MD –8.82 μmol/L, 95% CI –21.52 to 3.88; low certainty) nor resistance training (MD –10.15 μmol/L, 95% CI –29.47 to 9.17; low certainty) achieved statistically significant reductions versus usual care. The wide confidence interval observed for RT underscores the high imprecision inherent in indirect geometric pathways. These exploratory findings reinforce that demographic-adjusted eGFR remains the more reliable and unconfounded clinical indicator of renal adaptations to exercise.

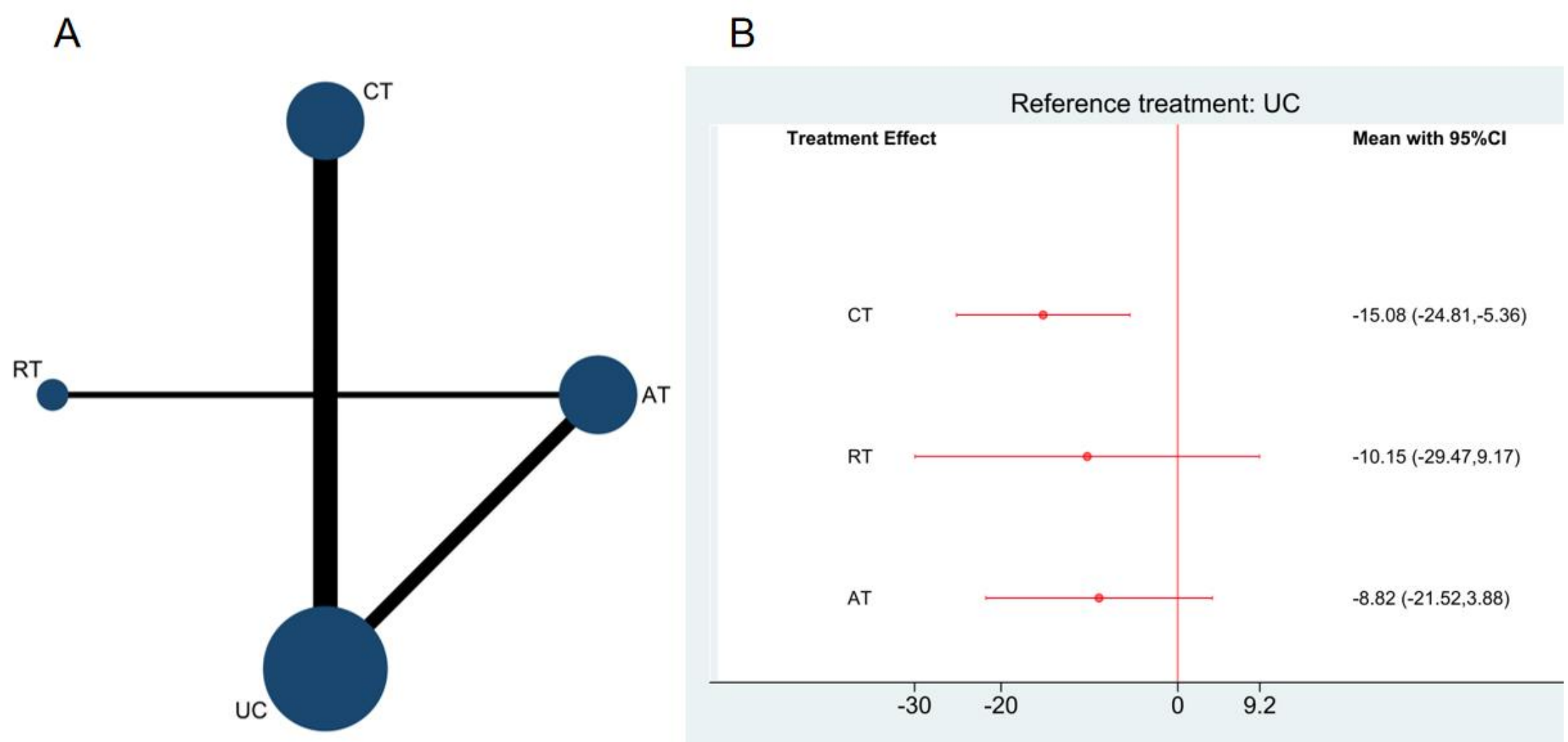


Note: In panel B, a mean difference (MD) < 0 indicates a favourable effect of the specific exercise modality in reducing SCr compared with usual care (UC). Node sizes in panel A reflect total sample sizes, and edge thicknesses correspond to the number of direct comparison trials.

Abbreviations: AT, aerobic training; CI, confidence interval; CT, combined training (aerobic plus resistance); MD, mean difference; RT, resistance training; SCr, serum creatinine; UC, usual care.
